# A Phylogeny-Informed Analysis of the Global Coral-Symbiodiniaceae Interaction Network Reveals that Traits Correlated with Thermal Bleaching Are Specific to Symbiont Transmission Mode

**DOI:** 10.1128/mSystems.00266-21

**Published:** 2021-05-04

**Authors:** Timothy D. Swain, Simon Lax, Jack Gilbert, Vadim Backman, Luisa A. Marcelino

**Affiliations:** a Department of Marine and Environmental Science, Nova Southeastern University, Dania Beach, Florida, USA; b Integrative Research Center, Field Museum of Natural History, Chicago, Illinois, USA; c Department of Ecology and Evolution, University of Chicago, Chicago, Illinois, USA; d Physics of Living Systems, Department of Physics, Massachusetts Institute of Technology, Cambridge, Massachusetts, USA; e Department of Pediatrics and Scripps Institution of Oceanography, University of California San Diego, La Jolla, California, USA; f Department of Biomedical Engineering, Northwestern University, Evanston, Illinois, USA; g Department of Civil and Environmental Engineering, Northwestern University, Evanston, Illinois, USA; University of Georgia

**Keywords:** coral bleaching, phylogeny, symbiotic network, transmission modes

## Abstract

The complex network of associations between corals and their dinoflagellates (family Symbiodiniaceae) are the basis of coral reef ecosystems but are sensitive to increasing global temperatures. Coral-symbiont interactions are restricted by ecological and evolutionary determinants that constrain partner choice and influence holobiont response to environmental stress; however, little is known about how these processes shape thermal resilience of the holobiont. Here, we built a network of global coral-Symbiodiniaceae associations, mapped species traits (e.g., symbiont transmission mode and biogeography) and phylogenetic relationships of both partners onto the network, and assigned thermotolerance to both host and symbiont nodes. Using network analysis and phylogenetic comparative methods, we determined the contribution of species traits to thermal resilience of the holobiont, while accounting for evolutionary patterns among species. We found that the network shows nonrandom interactions among species, which are shaped by evolutionary history, symbiont transmission mode (horizontally transmitted [HT] or vertically transmitted [VT] corals) and biogeography. Coral phylogeny, but not Symbiodiniaceae phylogeny, symbiont transmission mode, or biogeography, was a good predictor of thermal resilience. Closely related corals have similar Symbiodiniaceae interaction patterns and bleaching susceptibilities. Nevertheless, the association patterns that explain increased host thermal resilience are not generalizable across the entire network but are instead unique to HT and VT corals. Under nonstress conditions, thermally resilient VT coral species associate with thermotolerant phylotypes and limit their number of unique symbionts and overall symbiont thermotolerance diversity, while thermally resilient HT coral species associate with a few host-specific symbiont phylotypes.

**IMPORTANCE** Recent advances have revealed a complex network of interactions between coral and Symbiodiniaceae. Specifically, nonrandom association patterns, which are determined in part by restrictions imposed by symbiont transmission mode, increase the sensitivity of the overall network to thermal stress. However, little is known about the extent to which coral-Symbiodiniaceae network resistance to thermal stress is shaped by host and symbiont species phylogenetic relationships and host and symbiont species traits, such as symbiont transmission mode. We built a frequency-weighted global coral-Symbiodiniaceae network and used network analysis and phylogenetic comparative methods to show that evolutionary relatedness, but not transmission mode, predicts thermal resilience of the coral-Symbiodiniaceae holobiont. Consequently, thermal stress events could result in nonrandom pruning of susceptible lineages and loss of taxonomic diversity with catastrophic effects on community resilience to future events. Our results show that inclusion of the contribution of evolutionary and ecological processes will further our understanding of the fate of coral assemblages under climate change.

## INTRODUCTION

Symbiotic associations between reef-building corals and dinoflagellate microalgae (family Symbiodiniaceae) form the basis of the immense productivity and biodiversity of coral reefs. High taxonomic diversity in both partners (1–3) allows multiple coral-symbiont combinations with unique costs and benefits that may result in individual functional traits capable of determining fitness and the overall response of each unique holobiont to environmental stressors. These associations can be affected differentially by thermal stress through a variety of mechanisms determined by the traits of both partner species and the characteristics of their interactions (4). Their disassociation (bleaching) results in a range of impairments that culminate in coral mass mortality and ecosystem collapse (5). Unprecedented climate change-induced mass coral bleaching and death over the past decades (5) impart an urgency to understand how the diversity of associations effect resilience to thermal stress.

Our current understanding of species and interaction traits that affect differential bleaching are based on more than 20 years of observational and experimental data collected from a few species of either partner (the reductionist approach), and the detected patterns may or may not be generalizable across the ∼840 extant reef coral species (or some subset thereof) and their associated symbionts. Building data-informed hypotheses on the broad-scale effects of associating with thermotolerant Symbiodiniaceae (and correlates with any other species or interaction traits) can be accomplished through a systems approach that relies on the network of coral-Symbiodiniaceae interactions and simultaneously examines patterns across hundreds of coral and Symbiodiniaceae species and thousands of their symbiotic interactions. Coral-Symbiodiniaceae associations have been shown to form a complex network of interactions (6–8), similar to other mutualistic associations across the tree of life (e.g., plant-animal or macroorganisms-microbes [9–11]). Coral-Symbiodiniaceae networks are comprised of two types of nodes (coral species and symbiont phylotypes) which are linked together if an interaction has been observed. Similar to other mutualistic networks, such as plant-pollinator networks, coral-Symbiodiniaceae networks are very heterogeneous; most nodes have few links (i.e., specialists that associate with one or few partners [1, 2, 12, 13]), but some nodes have many links (i.e., generalists that associate with numerous partners [1, 2, 6, 12, 13]), providing connectivity to the broader network (7, 8).

Network analysis of coral-Symbiodiniaceae associations (6–8) has recently become possible due to multiple advances in (i) large-scale assemblage of ecological data sets with multiple species traits and biogeographic information (8, 14–16), (ii) higher-resolution sequencing (4), (iii) population genetic analysis (cf. reference 13), (iv) standardization of indices of thermal stress tolerance for both coral and Symbiodiniaceae partners (17, 18), and (iv) minimization of sampling and evolutionary biases in the meta-analysis of species interactions to reveal true dependences between partner association and holobiont traits (8). These initial network-based studies have advanced our understanding of coral-symbiont interactions and factors restricting their interactions (6, 8), and the effect of overall structure on network stability under environmental perturbations (7). Coral-symbiont interactions are constrained by both ecological and evolutionary processes, such as biogeographic distributions, locally adapted hosts and symbionts, and evolutionary constraints on partner choice (1, 2, 13, 19). Restrictions on species interactions structure the symbiosis network with a potential reduction in diversity of trait combinations and response strategies to environmental stress. For example, symbiont transmission mode (“vertical” if symbionts are packed in propagules by the parent or “horizontal” if they are environmentally acquired by the planula; reviewed in reference 20) has been found to structure the overall network into vertical or horizontal transmission subnetworks, each associated with a subset of unique phylotypes and a few generalist types, with mixed-mode types interacting with both (6). Since vertically transmitted (VT) Symbiodiniaceae appear to be more thermotolerant than horizontally transmitted (HT) Symbiodiniaceae (21), vertical transmission symbioses could exhibit enhanced resilience to thermal stress. The effect of overall structure of the network on its stability to perturbations was evaluated by Williams and Paterson (7), who mapped thermotolerance of both partners onto a global network of coral-Symbiodiniaceae associations and modeled their likelihood of bleaching (i.e., node and link removals) under thermal stress (i.e., increasing sea surface temperature thresholds known to cause bleaching). They found that local and global association patterns and environmental stress restrict interactions between coral and Symbiodiniaceae, resulting in increased sensitivity to thermal stress of the overall network.

However, it is not known how evolutionary histories of host species constrain the number or identity of the Symbiodiniaceae phylotypes they associate with and how these resulting trait combinations influence holobiont resilience to thermal stress. Coral-Symbiodiniaceae associations structured by phylogenetic relationships could reveal coral species associated with similar numbers or types of Symbiodiniaceae partners that are more closely related than expected by chance and exhibit similar bleaching susceptibility and fate during a thermal stress event (8). Several coral traits have shown phylogeny-associated patterns (e.g., coral coloniality and symbiosis, symbiont acquisition mode, light scattering in the coral skeleton, and partner specificity [20–24]), and phylogenetic structure has been observed in multiple networks with diverse ecological interactions (i.e., antagonistic or mutualistic) across the tree of life (9, 10, 25). Here, we ask to what extent coral-Symbiodiniaceae trait combinations determine thermal resilience in corals, while accounting for the number and type of associations that may be nonrandomly linked (i.e., phylogenetically related).

We compiled the largest data set of global coral-*Symbiodinium* associations yet assembled, comprising 152 reef-building corals and their 385 associated Symbiodiniaceae phylotypes observed under nonbleaching conditions, and built a frequency-weighted coral-symbiont interaction network. Evaluating interaction frequency between partners assumes that preferred and occasional interactions hold valuable ecological information that could result in different physiological responses of the holobiont (26), as opposed to assessing the presence or absence of interactions which assumes that all established associations are equally important (e.g., references 6 and 7).

We started by examining the network structure imparted by four factors known to restrict species interactions, namely symbiont transmission mode (6, 27), ocean biogeography (28, 29), life history strategy (competitive, weedy, stress tolerant, or generalist [14]), and shared ancestry (3, 29). We then evaluated whether the network structure imparted by these factors could explain gains in holobiont thermal resilience that result from associating with thermotolerant symbionts. We used recently standardized indices of thermotolerance for both Symbiodiniaceae (18) and coral (17). Specifically, we examined the effect of the number of potential symbiont partners (richness), their interdependence given other interactions (specificity), and preference of association with thermotolerant phylotypes (frequency of interaction) on the bleaching resilience of corals in the network. Although the ability to associate with thermotolerant phylotypes may allow corals to respond more effectively to thermal stress (adaptive bleaching hypothesis [30, 31]), generalist coral species have shown greater susceptibility to environmental stress, including thermal stress, than specialists (32, 33). Furthermore, the observed tradeoff between nutrition provision and thermotolerance (34) could limit functional diversity in symbiont assemblages. Therefore, we asked whether (i) corals with high richness or low specificity show lower thermal resilience, (ii) Symbiodiniaceae assemblages with higher mean thermotolerance (interaction frequency-weighted mean of thermotolerance scores [mean-TT]) and thermotolerance diversity (standard error [stderror] mean-TT) reduce coral bleaching response, and (iii) frequency of interaction with thermotolerant phylotypes under nonbleaching conditions, even if at low abundances (as a potential reservoir for future phylotype shifting), increase coral thermal resilience (see Table S1 in reference 35).

## RESULTS

### The coral-Symbiodiniaceae network exhibits high heterogeneity and modularity.

**FIG 1:**
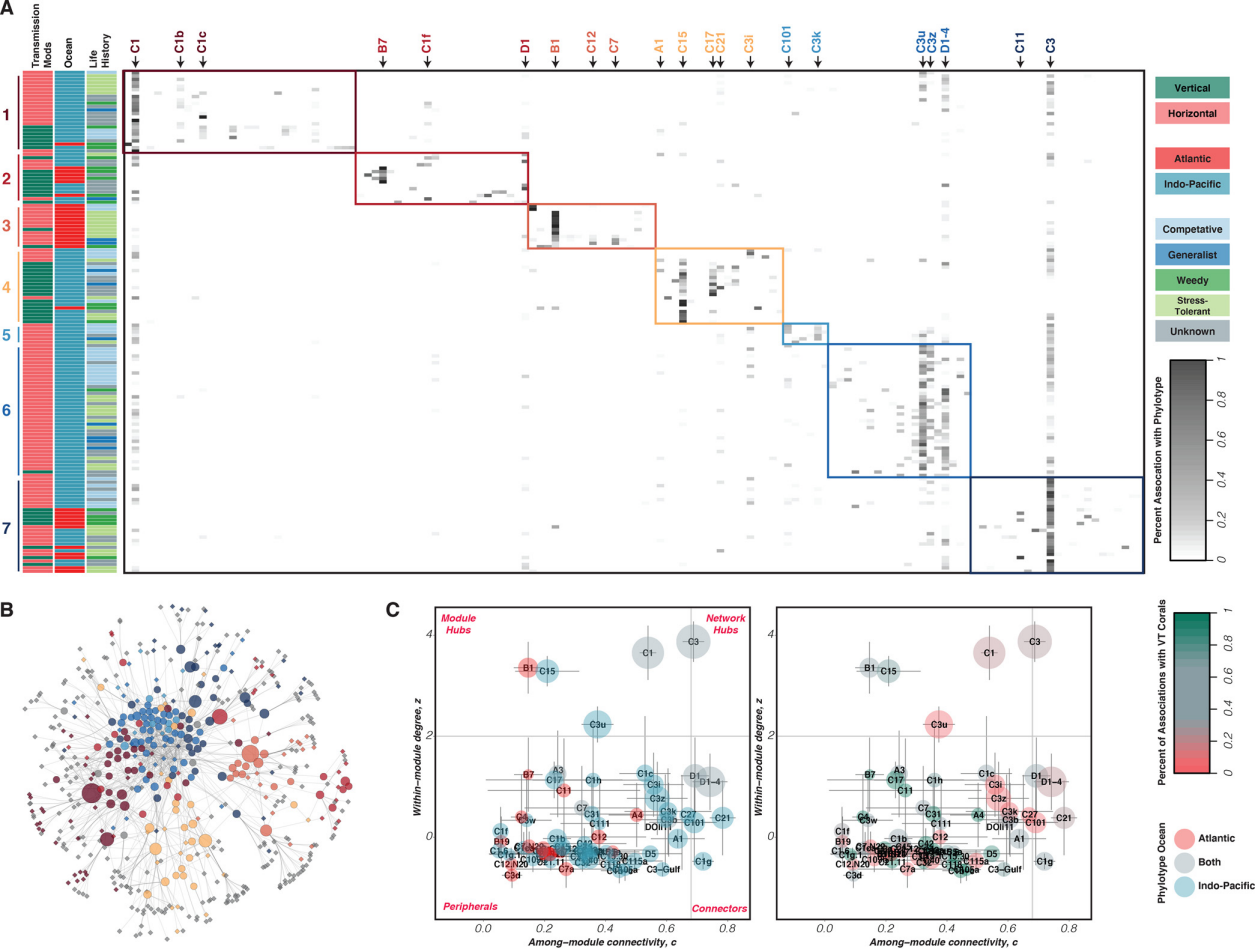
The coral-*Symbiodiniaceae* interaction network is significantly modular, highlighting the nonrandom interaction between hosts and their symbionts. (A) We used a weighted hierarchical random graph algorithm to uncover highly connected subnetworks (“modules”) within our full association network. These 7 modules, indicated by color blocks along the diagonal, were only weakly associated with coral transmission mode, geography, and life history (indicated by colored bars along the row). Select phylotypes, which play important roles in structuring the network, are indicated by arrows, with text color matched to module color. For a higher-resolution version of the heatmap, with all names included, see Fig. S3 in the supplemental material. (B) Module membership mapped onto the full interaction network, with colors as in panel A. Gray phylotypes were not included in the modularity analysis (but were included in all subsequent analyses) because they had less than 3 records in the data set. (C) The importance of individual phylotypes to the overall network structure can be assessed in terms of their among-module connectivity (*c*) and their within-module degree (*z*). Because module calculation is based on a random graph algorithm, we ran 100 different permutations, and points represent the average *c* and *z* values across those permutations. Error bars indicate the standard deviation (SD). Points represent individual phylotypes and are sized by their raw richness. Color represents the ocean in which the phylotypes are found (left) or the percentage of their associations that are with vertically transmitting corals (right). The many phylotypes with a *c* score of <0.05 and a negative *z* score are excluded from the plot for clarity.

We built a frequency of interaction matrix between 152 coral species and 385 Symbiodiniaceae phylotypes (see Tables S3 to S5 in reference 35), which was represented as a bipartite network in which a few generalist phylotypes and coral species create a cohesive central core of interconnections, while most phylotypes and corals are specialists located at the periphery of the network (Fig. 1B; see also Fig. S2 in the supplemental material). This network exhibited high heterogeneity, with some species interacting more often than expected by chance, few realized interactions (connectance = 0.0145), more phylotypes than coral species (asymmetry = −0.436), and no one-to-one exclusive associations (see Fig. S1 in the supplemental material; see also Table S3 in reference 35). The network was significantly modular (modularity index [Q] = 0.568, 333 standard deviations above random), comprising seven identified modules, which were structured to various degrees by the mode of symbiont transmission, ocean basin, and life-history strategy (Fig. 1A; see also Fig. S3 in the supplemental material. After assigning within-module degree (*z*) and among-module connectance (*c*), the vast majority of phylotypes were identified as peripheral nodes or specialists (low *c* and *z* scores) with very few overall links, predominantly to coral species within their modules (e.g., Cladocopium C3z; Fig. 1C). A small number of generalist phylotypes were identified as module hubs (low *c* and high *z*), which are highly connected within their modules but are not often connected to other modules (e.g., Cladocopium C1 or Cladocopium C15; Fig. 1C), and module connectors (high *c* and low *z*), which link modules together into a cohesive network (e.g., Durusdinium trenchii or Durusdinium D1-4 and *Cladocopium* C21; Fig. 1C). Only one phylotype was identified as a network hub or super-generalist, with common associations across modules (*Cladocopium* C3; Fig. 1C).

10.1128/mSystems.00266-21.1 FIG S1 Bipartite graph of the simplified coral-Symbiodiniaceae interaction network, showing high heterogeneity and asymmetry among species and phylotypes. Coral species and phylotypes are represented by a block. For clarity, only corals with at least 50 records of association and phylotypes with at least 10 records of association are included in the graph. Links connecting species are shown as lines with different thickness that are proportional to link strength (i.e., their number of associations). The lines connecting corals and phylotypes that share associations have widths that correspond to the total number of those pairwise associations (not the percentage of that coral’s associations, as in the network visualizations). Download FIG S1, PDF file, 0.04 MB. Copyright © 2021 Swain et al. 2021 Swain et al. https://creativecommons.org/licenses/by/4.0/ This content is distributed under the terms of the Creative Commons Attribution 4.0 International license.

10.1128/mSystems.00266-21.2 FIG S2 (A) Symbiodiniaceae internal transcribed spacer 2 of the ribosomal RNA nuclear gene (ITS2 rDNA) genetic types (385 phylotypes) nodes key in the coral-Symbiodiniaceae interaction network (see also Tables S2 and S4 in reference 35). (B) Coral species nodes key in the coral-Symbiodiniaceae interaction network (see also Table S2 and S3 in reference 35). Download FIG S2, PDF file, 3.3 MB. Copyright © 2021 Swain et al. 2021 Swain et al. https://creativecommons.org/licenses/by/4.0/ This content is distributed under the terms of the Creative Commons Attribution 4.0 International license.

10.1128/mSystems.00266-21.3 FIG S3 High-resolution version of Fig. 1A. The coral-Symbiodiniaceae interaction network has a modular organization. (A) A weighted hierarchical random graph algorithm identifies 7 modules in the network, with the 152 coral species represented by rows and the 136 phylotypes with >3 records indicated by columns. Modules are indicated by the color blocks along the diagonal, and three coral metadata factors (transmission mode, ocean, and life history strategy) are indicated by colored bars along the row. Select phylotypes, which play important roles in structuring the network, are indicated by arrows, with text color matched to module color. Download FIG S3, PDF file, 0.8 MB. Copyright © 2021 Swain et al. 2021 Swain et al. https://creativecommons.org/licenses/by/4.0/ This content is distributed under the terms of the Creative Commons Attribution 4.0 International license.

### The coral-Symbiodiniaceae network is structured to various degrees by mode of symbiont transmission, ocean biogeography, and life-history strategy.

**FIG 2:**
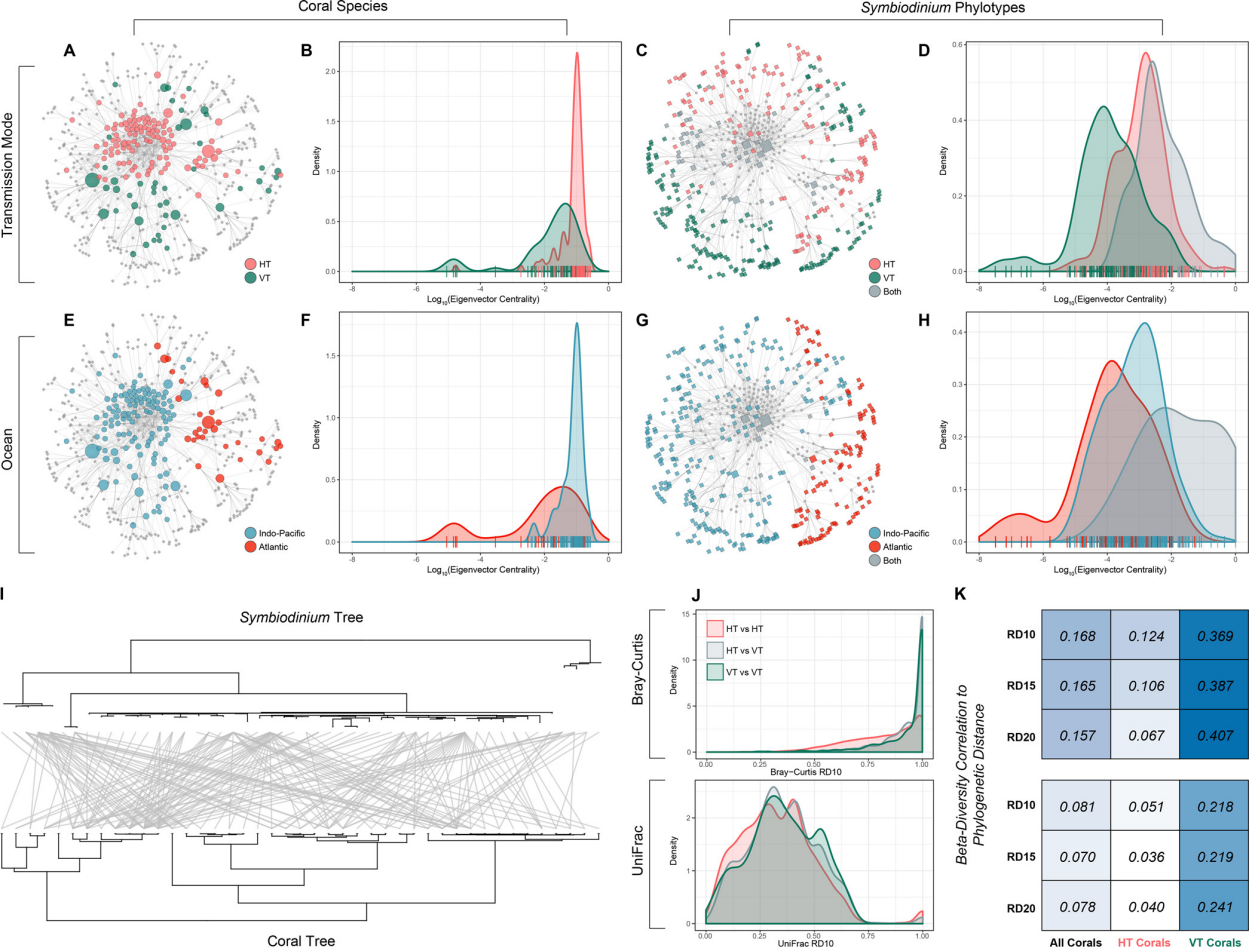
Transmission mode and geography structure the coral-*Symbiodiniaceae* interaction network. (A to D) Corals which transmit their symbionts horizontally, and phylotypes which are transmitted horizontally, are significantly more central in the interaction network. (A) Coral nodes, sized by rarefied richness (RD_10_), colored by their phylotype transmission mode. (B) Density plots of eigenvector centrality for coral species, grouped by transmission mode. Individual data points are indicated by tick marks along the *x* axis. (C) Phylotype nodes are colored by their transmission mode and sized by their raw richness (see section S.7 in reference 35). (D) Density plots of the eigenvector centrality of Symbiodiniaceae phylotypes, grouped by their transmission mode. (E to H) Indo-Pacific corals, and symbionts which occur in both ocean basins, are the most central within the interaction network. Panels are as in panels A to D, grouped by ocean rather than by transmission mode. (I) There is no strong association between the phylogeny of corals and that of their phylotypes. Tanglegram of the Symbiodiniaceae (top) and coral (bottom) phylogenies for vertically transmitting corals and their associated phylotypes. Edges connect phylotypes to the corals they share associations within the data set. (J) Transmission mode has little effect on the similarity of symbiont partners between coral species. Density plots of the beta diversity between coral species, grouped by whether the comparison is between two corals of the same transmission mode or of different transmission mode. Beta diversity is calculated as Bray Curtis dissimilarity (top), which is a weighted but nonphylogenetic metric, or unweighted UniFrac distance (bottom), which is phylogeny based. (K) Closely related corals are more likely to have similar symbiont partners than more distantly related corals, but this trend is driven almost entirely by vertically transmitting corals. Heatmap of the Pearson correlations between beta diversity and phylogenetic distance, with a darker color indicating a higher correlation. The top heatmap uses Bray-Curtis as the beta diversity metric, and the bottom uses UniFrac. Within each heatmap, three rarefaction depths are represented by the rows, and three species groups (all corals, only HT corals, and only VT corals) are represented by columns.

The constraints imposed by symbiont transmission mode and ocean biogeography resulted in deeply partitioned subnetworks (ecologically defined sections of the global network that are visibly isolated; Fig. 2A to H). HT corals and their uniquely associated symbionts are highly connected in the center of the network (as measured by eigenvector centrality) while VT corals and their uniquely associated symbionts were predominantly located toward the periphery (Fig. 2A to D, phylogenetic logistic regression [PLR] no. 1, *P = *0.027; see also Table S6 in reference 35). Atlantic and Indo-Pacific subnetworks, with their respective phylotypes, were not significantly different in centrality (Fig. 2E to H, PLR no. 2, *P = *0.333; see also Table S6 in reference 35), possibly due to a smaller subset of Atlantic coral species and phylotypes in our data set relative to the Indo-Pacific data (32 versus 120 coral species and 130 versus 265 phylotypes). Mixed-mode-transmitted (MMT) phylotypes and phylotypes found in both oceans are the most central in the network and connect both HT and VT subnetworks and Atlantic and Pacific subnetworks, respectively (Fig. 2C and D and G and H, respectively, phylogenetic analysis of variance [PANOVA] no. 1 and 2, *P = *0.006 to 0.02; see also Table S7 in reference 35). Differences in life history strategies did not have clear structural effects on the network (see Fig. S4 in the supplemental material, PANOVA no. 3, *P = *0.703; see also Table S7 in reference 35).

10.1128/mSystems.00266-21.4 FIG S4 Coral life history strategies mapped onto the coral-Symbiodiniaceae interaction network. Download FIG S4, PDF file, 0.8 MB. Copyright © 2021 Swain et al. 2021 Swain et al. https://creativecommons.org/licenses/by/4.0/ This content is distributed under the terms of the Creative Commons Attribution 4.0 International license.

Further characterization of species interactions within HT and VT subnetworks revealed that the number of potential links (rarefied-richness; see Fig. S5 in the supplemental material) and the relative strength of the link of a coral-phylotype pair relative to all link strengths of that phylotype (specificity *d*′; Fig. S5) did not significantly differ between HT and VT corals (PLR no. 3 and 4, *P = *0.556 to 0.783; see also Table S6 in reference 35), nor were they correlated with each other for either subnetwork (phylogenetic generalized least-squares regression [PGLS] no. 1 and 2, *P = *0.071 to 0.061; see also Table S8 in reference 35).

10.1128/mSystems.00266-21.5 FIG S5 Rarefied richness and the relative strength of the link of a coral-phylotype pair relative to all link strengths of that phylotype (specificity *d*′) mapped onto the coral-Symbiodiniaceae interaction network. Download FIG S5, PDF file, 1.8 MB. Copyright © 2021 Swain et al. 2021 Swain et al. https://creativecommons.org/licenses/by/4.0/ This content is distributed under the terms of the Creative Commons Attribution 4.0 International license.

### The phylogenies of both corals and Symbiodiniaceae predict their interaction patterns.

We evaluated whether association patterns are partially dependent on phylogenetic relatedness (Fig. 2I), and we observed strong phylogenetic signals across scales. Coral species in the same module are significantly more closely related than species in different modules (nonparametric *t* test, *P < *10^−4^; see Fig. S6 in the supplemental material), and the same is true of phylotypes (nonparametric *t* test, *P < *10^−4^; Fig. S6). At the network scale, coral species exhibiting the same transmission mode (in either HT or VT subnetworks) are more closely related to each other than other corals, particularly within VT corals nonparametric *t* test, *P < *10^−4^; see Fig. S7 in the supplemental material.

10.1128/mSystems.00266-21.6 FIG S6 Nodes in the same module are significantly more phylogenetically related than nodes in different modules for both coral species and Symbiodiniaceae phylotypes. The left panels represent the distributions of phylogenetic distances for pairwise within-module and between-module comparisons. The right panels plot the distribution of *t* test statistics for 10,000 random module permutations, with the *t* test statistic for the observed modules indicated by the red point. Download FIG S6, PDF file, 0.7 MB. Copyright © 2021 Swain et al. 2021 Swain et al. https://creativecommons.org/licenses/by/4.0/ This content is distributed under the terms of the Creative Commons Attribution 4.0 International license.

10.1128/mSystems.00266-21.7 FIG S7 Corals with the same symbiont transmission mode are significantly more phylogenetically related than corals with different symbiont transmission modes (horizontally transmitted [HT] or vertically transmitted [VT]). The left panel represents the distributions of phylogenetic distances for pairwise comparisons between two VT corals, two HT corals, and two corals with different transmission modes. The right panel plots the distribution of *t* test statistics for 10,000 random permutations of transmission mode assignments, with the *t* test statistic for the correct transmission mode assignments indicated by the red point. Download FIG S7, PDF file, 0.3 MB. Copyright © 2021 Swain et al. 2021 Swain et al. https://creativecommons.org/licenses/by/4.0/ This content is distributed under the terms of the Creative Commons Attribution 4.0 International license.

Furthermore, as phylogenetic distances between corals increases, Bray-Curtis (dissimilarity in phylotype proportion and composition; Fig. 2J) and UniFrac (phylogenetic distance between communities; Fig. 2J) indices become greater, especially in VT corals, indicating that more closely related coral species associate with similar sets of phylotypes sampled from similar regions of the Symbiodiniaceae phylogeny (i.e., phylosymbiosis). To confirm that the rank ordering of diversity was independent of the rarefaction level, the estimates of richness and specificity were repeated using subsets of coral species with increasingly high rarefaction levels (rarefaction depth of 10, 15, and 20 interaction records per coral [RD_10_, RD_15_, and RD_20]_ subsets), which yielded stable trends of Bray-Curtis and UniFrac dependence on the phylogenies of both partners (Fig. 2K; see also “Robustness of our results”).

### Coral phylogeny, but not Symbiodiniaceae phylogeny, symbiont transmission, ocean biogeography, or life history strategies, is a good predictor of thermal tolerance.

**FIG 3:**
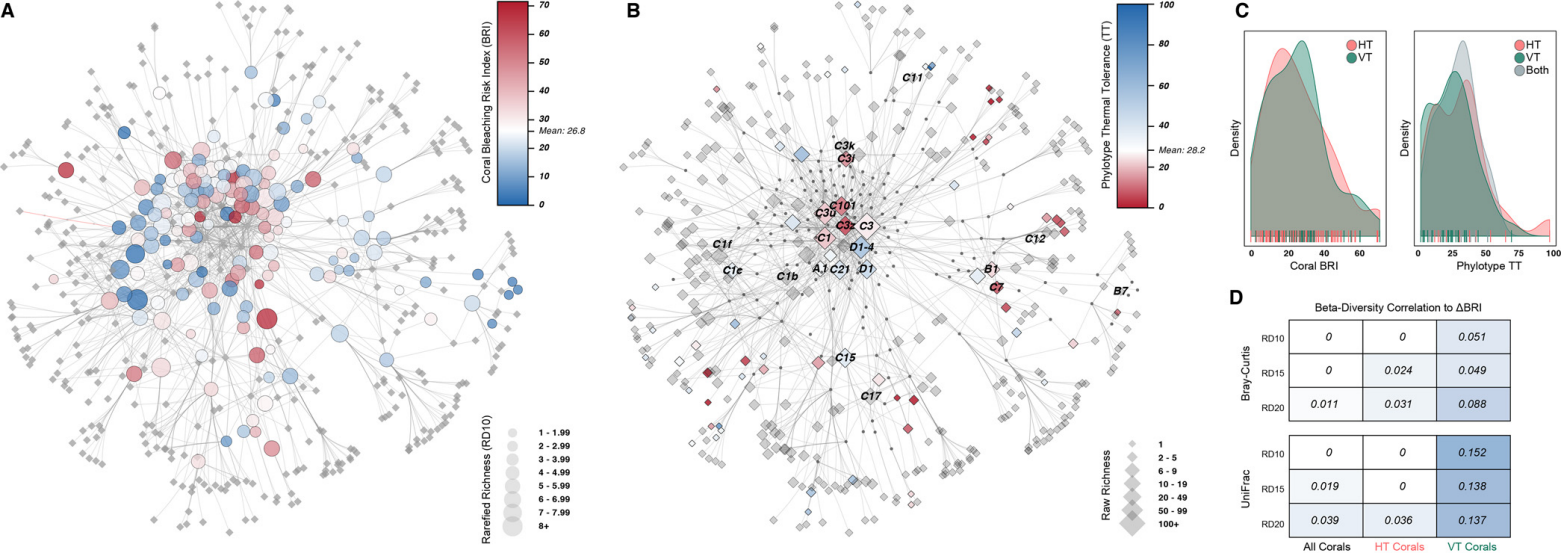
Network of coral bleaching index (taxon-BRI) and Symbiodiniaceae thermal tolerance (TT). (A) Coral nodes, sized by rarefied richness (RD_10_) and colored by taxon-BRI. (B) Phylotype nodes, sized by raw richness and colored by TT. Phylotypes indicated by name in Fig. 1A are labeled. (C) Density plots of coral taxon-BRI (left) and phylotype TT (right), grouped by transmission mode. Individual data points are indicated by tick marks along the *x* axis. (D) Heatmap of the Pearson correlation between beta diversity and ΔBRI, with organization identical to that of Fig. 2J. Correlations of <0.01 are reduced to 0.

Coral bleaching response index (taxon-specific bleaching response index [taxon-BRI]) and phylotype thermotolerance (TT) scores showed high variability among coral species (mean ± standard deviation [SD], 26.8 ± 17.4; *n* = 152) and Symbiodiniaceae phylotypes (28.8 ± 18.2; *n* = 73; Fig. 3A and B; see also Tables S2 and S3 in reference 35). However, they were not significantly structured across the entire network (PGLS no. 3 and 4, *P = *0.152 and 0.905; see Table S8 in reference 35), nor could we detect different bleaching responses between VT and HT corals (Fig. 3C, PLR no. 5 *P = *0.5; see also Table S6 in reference 35) or different thermotolerance between VT and HT phylotypes (Fig. 3C, PANOVA no. 4, *P = *0.935; see also Table S7 in reference 35). In addition, no significant differences in taxon-BRI were found among the four life history strategies (PANOVA no. 5, *P = *0.7; see also Table S7 in reference 35), nor among corals across ocean basins (PLR no. 22, *P = *0.988; see also Table S6 in reference 35), nor was thermotolerance of their symbionts different across ocean basins (PLR no. 23, *P = *0.972; see also Table S6 in reference 35). These results demonstrate a uniform distribution of susceptible and resilient coral species and phylotypes across both the network and mode-of-transmission-defined subnetworks.

Coral phylogeny was found to be a good predictor of bleaching susceptibility; it was able to explain 64% of the variation in bleaching susceptibility in the overall network (phylogenetic signal [Pagel’s λ] no. 1, *P = *0.005; see also Table S9 in reference 35), and 42% and 58% in the VT and HT subnetworks (Pagel’s λ no. 2 and 3, VT *P = *0.048 and HT *P = *0.019; see also Table S9 in reference 35). However, thermotolerance could not be inferred from the Symbiodiniaceae phylogeny either across the network (Pagel’s λ no. 4, *P = *0.148; see also Table S9 in reference 35) or transmission subnetworks (Pagel’s λ no. 5 and 6, VT *P = *0.34 and HT *P* =1; see also Table S9 in reference 35). Furthermore, VT corals with similar bleaching susceptibility were more likely than HT corals to associate with Symbiodiniaceae assemblages of similar composition and phylogenetic relatedness (Bray-Curtis and UniFrac; Fig. 3D). Significant, but weak, correlations were observed for HT corals only when subsets of species with greater sampling intensity (i.e., RD_20_) were tested.

### Symbiodiniaceae assemblages with higher mean thermotolerance do not reduce coral bleaching susceptibility; thermotolerance diversity increases susceptibility only in VT corals.

**FIG 4:**
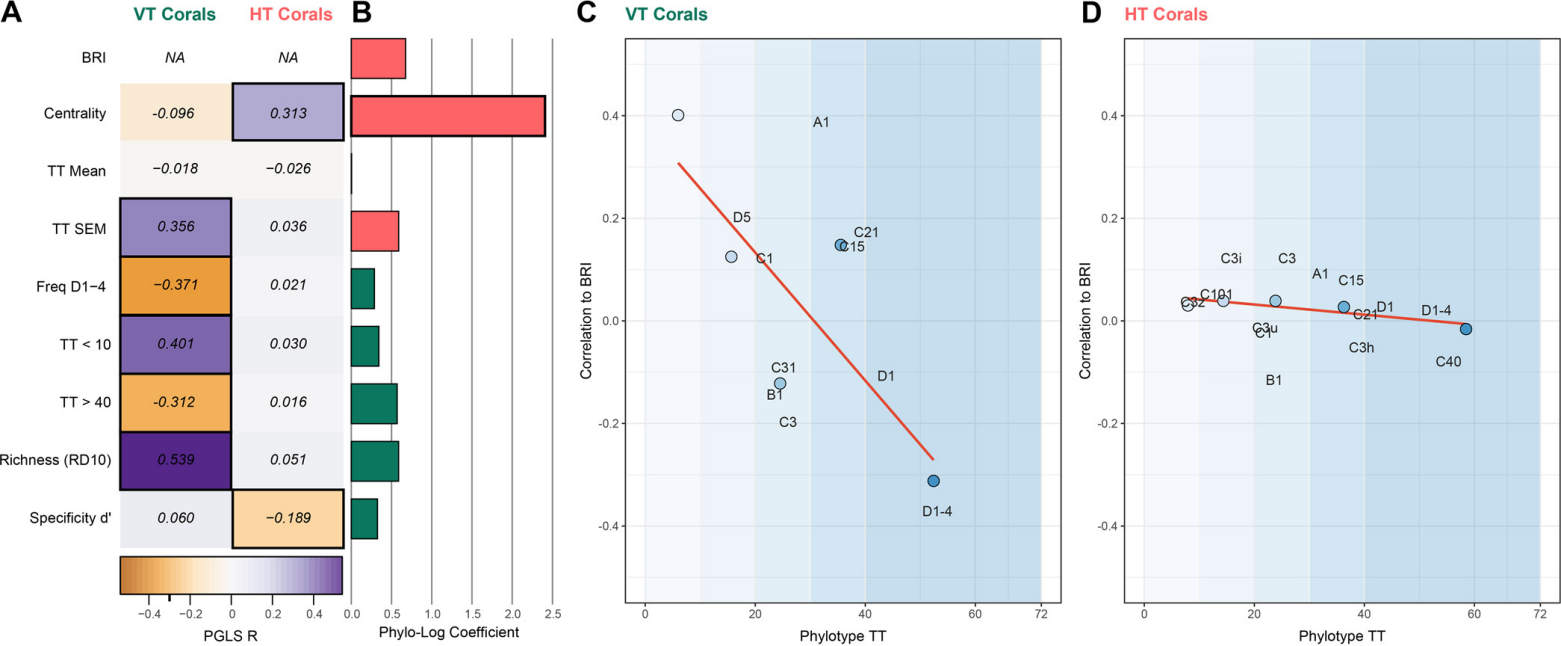
Effect of transmission mode on Symbiodiniaceae associations. (A) Heatmap of phylogenetic generalized least-squares regression coefficients (PGLS *R*) between coral metrics (“Freq D1-4” means frequency of association with Durusdinium trenchii or Durusdinium D1-4) and taxon-BRI within the VT (left) and HT (right) subnetworks. Positive values (purple) indicate that larger values of that variable are associated with higher bleaching risk (taxon-BRI). Significant regressions are indicated by black borders around the heatmap cell. (B) Bar chart of phylogenetic logistic regression values for traits against transmission mode. Values indicate the extent to which variance in the trait is associated with transmission mode, and bar color indicates whether higher values of the trait are more associated with HT corals (pink) or VT corals (green). Significant associations (centrality only) are indicated by a thicker black border around the bar. (C) Associating with phylotypes in different thermotolerance groups has variable effects on taxon-BRI in VT corals. Phylotypes were grouped into 5 TT bins, whose boundaries are indicated by the blue color blocks. Points represent the average TT of phylotypes in each bin (*x* axis) and the PGLS *R* between the percentage of coral associations that are with phylotypes in that bin against taxon-BRI (*y* axis). Values for select individual phylotypes are indicated by the phylotype name, and the orange line is the correlation between the *x* and *y* axes for the five individual points. (D) As in panel C, but for HT corals.

Mean thermotolerance (mean-TT) of the Symbiodiniaceae assemblage was not structured across the network (PGLS no. 5, *P = *0.434; see also Table S8 in reference 35), did not differ between HT and VT corals (Fig. 4B, PLR no. 6, *P = *0.997; see also Table S6 in reference 35) and showed no correlation with taxon-BRI for either of the transmission subnetworks (Fig. 4A, PGLS no. 6 and 7, *P = *0.796 and 0.909; see also Table S8 in reference 35). Importantly, the mean-TT of high-BRI corals did not differ from that of low-BRI corals (PLR no. 7, *P = *0.06; see also Table S6 in reference 35).

Thermotolerance diversity (stderror mean-TT) was not structured across the network (PGLS no. 8, *P = *0.776; see also Table S8 in reference 35), and, although higher in high-BRI corals (PLR no. 8, *P* = 0.009; see also Table S6 in reference 35), it was not significantly different between HT and VT subnetworks (Fig. 4B, PLR no. 9, *P = *0.556; see also Table S8 in reference 35). Nevertheless, taxon-BRI was positively correlated with the diversity of thermotolerance of Symbiodiniaceae assemblages across the VT subnetwork (Fig. 4A, PGLS no. 9, *P = *0.019; see also Table S8 in reference 35) but not the HT subnetwork (Fig. 4A, PGLS no. 10, *P = *0.713; see also Table S8 in reference 35).

Frequency of interaction with thermotolerant phylotypes under nonbleaching conditions is correlated with coral thermal resilience in VT corals but not in HT corals. No differences in frequency of interaction with phylotypes in any of the thermotolerance groups, nor with thermotolerant D. trenchii, were observed between VT and HT corals (Fig. 4A and B and Fig. S8, PLR no. 10 to 15, *P* = 0.732 to 0.775; see also Table S6 in reference 35) and for the most part between high-BRI and low-BRI corals (PLR no. 16 to 21, *P* = 0.063 to 0.179; see also Table S6 in reference 35). However, taxon-BRI of VT corals was positively related to the frequency of interaction with low thermotolerance Symbiodiniaceae (Fig. 4C, TT < 10, PGLS no. 11 and 12, *P* values = 0.006; see also Tables S8 and S10 in reference 35) and negatively correlated with the frequency of interaction with high thermotolerance Symbiodiniaceae (Fig. 4C, TT > 40, PGLS no. 13 and 14, *P* = 0.035 and 0.039; see also Table S8 in reference 35), but were not significantly affected by the frequency of interaction with moderate thermotolerance phylotypes (Fig. 4C, 10 < TT ≤ 40, PGLS no. 15 to 20, *P* = 0.406 to 0.398; see also Table S8 in reference 35). The correlation between frequency of interaction with individual phylotypes of known thermotolerance and taxon-BRI followed the trend of the overall correlation (Fig. 4C, PGLS no. 21 to 31, *P* = 0.178 to 0.25; see also Table S8 in reference 35). Unexpectedly, frequency of interaction with thermotolerant phylotypes was not correlated with the bleaching response of HT corals (Fig. 4D, PGLS no. 32 and 33, *P* = 0.873 and 0.908; see also Tables S8 and S10 in reference 35), nor was frequency of interaction with thermosensitive phylotypes (Fig. 4D, PGLS no. 34 and 35, *P* = 0.758 and 0.639; see Table S8 in reference 35). In fact, the overall trend across all thermotolerance groups and individual phylotypes associated with HT corals was weak and not significant (Fig. 4D, PGLS no. 36 to 56, *P* = 0.691 to 0.422; see also Tables S8 and S10 in reference 35). Taken together, these results demonstrate that vertically transmitting corals draw clear benefits to cope with thermal stress from associating with thermotolerant phylotypes during nonbleaching conditions, while horizontally transmitting corals do not.

10.1128/mSystems.00266-21.8 FIG S8 Corals with different mode of transmission have similar phylotype frequency of association over a wide range of thermotolerance (5 thermotolerance [TT] bins, TT ≤ 10, 10 < TT ≤ 20, 20 < TT ≤ 30, 30 < TT ≤ 40, and TT > 40). Mean frequency of interaction ± standard error of the mean (SEM). Download FIG S8, PDF file, 0.1 MB. Copyright © 2021 Swain et al. 2021 Swain et al. https://creativecommons.org/licenses/by/4.0/ This content is distributed under the terms of the Creative Commons Attribution 4.0 International license.

### High symbiont richness increases bleaching susceptibility in VT but not HT corals; symbiont specificity does not affect bleaching susceptibility in corals.

Phylotype rarefied richness did not differ significantly between HT and VT subnetworks (Fig. 4B, PLR no. 3, *P = *0.556; see also Table S6 in reference 35). However, taxon-BRI of VT corals was positively correlated with rarefied richness (Fig. 4A, PGLS no. 57, *P < *0.001; see also Table S8 in reference 35) but taxon-BRI of HT corals was not (Fig. 4A, PGLS no. 58, *P = *0.604; see also Table S8 in reference 35), indicating that resilient VT corals limit the number of symbiont partners they associate with.

Specificity *d′* did not differ significantly between HT and VT subnetworks (Fig. 4B, PLR no. 4, *P = *0.783; see also Table S6 in reference 35), and taxon-BRI of VT corals showed no relationship to specificity *d′* (Fig. 4A, PGLS no. 59, *P = *0.69; see also Table S8 in reference 35). However, taxon-BRI of HT corals tended to decrease with specificity *d′*, but this trend was marginally significant (Fig. 4A, PGLS no. 60, *P = *0.053; see also Table S8 in reference 35), suggesting that resilient HT corals associate with a few host-specific phylotypes. The thermal resilience acquired by corals from associating with specialist phylotypes is not due to greater thermotolerance, since both richness and specificity *d′* of Symbiodiniaceae are unrelated to thermotolerance (PGLS no. 61 and 62, *P = *0.828 and 0.766, respectively; see also Table S8 in reference 35).

### Robustness of our results.

We tested whether using the internal transcribed spacer 2 of the ribosomal RNA nuclear gene (ITS2 rDNA) with known limitations, such as intragenomic variation (IGV) and low sensitivity to resolve host-specific lineages, could influence our findings. We built a new coral-Symbiodiniaceae matrix implementing the “metahaplotype” concept with 184 host-specific sequences identified as putative IGVs and ancestral phylotypes with seven or more IGVs and known thermotolerance (*Cladocopium* C1, *Cladocopium* C3, *Cladocopium* C15, *Cladocopium* C21, Breviolum B1, and Durusdinium D1), resulting in 293 unique coral-Symbiodiniaceae terminal nodes (Fig. S9; see also supplemental methods and Tables S3 and S11 in reference 35). In this modified network, we treated potential IGVs cooccurring with ancestral phylotypes in a single host as a single host-specific Symbiodiniaceae lineage metaphylotype. Although this approach eliminated about 25% of the unique associations from our network, it had a negligible effect on the main findings regarding rarefied richness, specificity, and taxon-BRI (PGLS 1 to 4, *P = *0.001 to 0.002; see also Table S12 in reference 35). Removal of links and nodes under specific constraints allows evaluation of the robustness of a network to extinction events or environmental attacks, such as thermal stress (7, 9, 36). In particular, Williams and Patterson (7) observed a 20% reduction in robustness of their coral-Symbiodiniaceae network when links were removed following their bleaching model (i.e., coral-symbiont links of known thermotolerance were removed as temperature thresholds were exceeded), but the network was robust to random link removals, suggesting that the heterogenous structure of the coral-Symbiodiniaceae network decreases its robustness to thermal stress. However, as more host-specific phylotypes (isolated nodes) are identified (see references 1, 37, and 38; see references 39 and 40 on the flexibility of coral-Symbiodiniaceae associations), it will be important to reevaluate the role of the coral-symbiont network of interactions on coral thermal resilience.

10.1128/mSystems.00266-21.9 FIG S9 Coral-Symbiodiniaceae network that includes metahaplotypes (152 coral species and 285 phylotypes, of which 66 are metahaplotypes) is similarly structured by ocean basin (Atlantic or Indo-Pacific) and symbiont mode of transmission (horizontal or vertical transmission) as the coral Symbiodiniaceae network between 152 coral species and 385 phylotypes (see Fig. 2). Potential intragenomic variants (IGV) cooccurring with ancestral phylotypes in a single host were treated as a single host-specific Symbiodiniaceae lineage metahaplotype (*sensu* Smith et al. [56]). Potential intragenomic variations (IGVs) without evidence of ancestral cooccurrence were eliminated from the dataset (see also section S.7, “Sensitivity analysis,” and Tables S4 and S11 in reference 35). Download FIG S9, PDF file, 2 MB. Copyright © 2021 Swain et al. 2021 Swain et al. https://creativecommons.org/licenses/by/4.0/ This content is distributed under the terms of the Creative Commons Attribution 4.0 International license.

We further tested whether sampling intensity could be a confounding factor in our analyses (8). We found that all metrics that correlated with taxon-BRI were independent of sampling intensity for both transmission subnetworks (PGLS no. 5 to 15, *P = *0.641 to 0.075; see also Table S13 in reference 35), except rarefied richness, which showed a small dependence on sampling intensity in VT (but not HT) corals (PGLS no. 16 to 21, *P = *0.013 to 0.997; see also Table S13 in reference 35). However, taxon-BRI was independent of sampling intensity for both VT and HT corals, demonstrating that the most densely sampled corals had both high and low thermal resilience (PGLS no. 22 and 23, *P = *0.116 and 0.916; see also Table S13 in reference 35). Furthermore, our findings were also retained in data sets with progressively greater accuracy in types and strength of interactions (subsets RD_10_, RD_15_, and RD_20_; PGLS no. 24 to 51, *P = *0.0268 to 0.413; see also section S.7 and Table S13 in reference 35). These results support the validity of the main findings and stress the need for evaluation of the biases involved in determining richness of phylotypes in corals.

## DISCUSSION

Here, we developed a systems approach to coral bleaching based upon network and phylogenetic comparative analyses to examine the connection between coral and Symbiodiniaceae species traits and thermal resilience.

We confirmed previous observations that most coral-symbiont interactions are specific, with a few phylotype generalists creating a nonrandom network of interactions (6, 7, 36, 41) that are structured by symbiont transmission mode (6) and ocean biogeography (7, 41). We found that coral-Symbiodiniaceae interactions are also structured by evolutionary histories of both coral and Symbiodiniaceae, indicating that more closely related coral species associate with similar sets of phylotypes sampled from similar regions of the Symbiodiniaceae phylogeny. However, only coral phylogeny, and not Symbiodiniaceae phylogeny, symbiont transmission mode, or biogeography, was a strong predictor of coral thermal resilience. Coral phylogeny was able to explain 64% of the variation in bleaching susceptibility in the overall network, which signifies that more closely related coral species exhibit similar bleaching susceptibility. These results are congruent with previews observations that bleaching susceptibility is partially determined by evolutionary relatedness (3, 21, 23). Conversely, Symbiodiniaceae thermotolerance is not genus (i.e., clade) specific and apparently evolved multiple times independently (24, 42). Our results underscore the importance of accounting for phylogenetic processes that may influence coral-Symbiodiniaceae symbiosis and their response to environmental stress. Standard phylogenetic comparative methods are widely applied to mutualistic networks (9, 10, 25), and are starting to be applied to coral-Symbiodiniaceae interactions (8), as phylogenies of both partners are better understood (3, 29). As a consequence of phylogenetic forces shaping interaction patterns, increased magnitude in frequency and intensity of thermal stress events could result in nonrandom pruning of susceptible lineages and loss of taxonomic diversity (3), with catastrophic effects on community resilience to future events. Similar fates were predicted for related species in a phylogenetically structured plant-animal mutualistic network when extinction events were simulated (9).

We examined the contribution of three ecological determinants to the holobiont thermal resilience, regardless of whether they significantly restricted coral-Symbiodiniaceae associations (ocean biogeography and symbiont transmission) or not (life history strategies). Both bleaching susceptibility and thermotolerance indices assigned to coral and Symbiodiniaceae nodes exhibited high variability among species (17, 18) and were not significantly different among ocean basins, symbiont transmission modes, or life history strategies. This uniform distribution of resilient and susceptible species across the network indicates that although some ecological determinants restrict the interactions among partners, they do not provide holobionts with increased benefits in thermal resilience.

To determine if specific interactions result in trait combinations that increase holobiont thermal resilience, we measured association with thermotolerant phylotypes (frequency of interaction, or link strength), overall thermotolerance of the associated phylotypes (both mean and diversity), richness of associations (number of unique partners or links), and specificity of associations (relative dependence on a given partner, or relative link strength). While certain patterns of associations were found to increase holobiont thermal resilience, they were not generalizable across the entire network and were instead constrained by symbiont transmission mode. However, the gains in thermal resilience observed from specific patterns of coral-Symbiodiniaceae association did not result in significant differences in thermal resilience between both groups, as both HT and VT corals exhibited a similarly wide range of bleaching responses. We show that under nonbleaching conditions, vertically transmitting corals may increase thermal resiliency by associating with thermotolerant phylotypes, reducing the thermotolerance diversity of their symbiont assemblage, or interacting with fewer phylotypes. Conversely, horizontally transmitting corals do not seem to associate preferentially with known thermotolerant phylotypes under normal conditions, but instead associate with few phylotypes that are host specific. Differences in bleaching resilience strategies might be due to contrasting symbiont acquisition strategies as predicted by symbiosis theory. Corals with vertical transmission inherit symbiont genotypes; this fidelity may better align the fitness interests of mutualist partners, but may be insufficiently flexible to cope with variable environmental stressors (11, 30, 43, 44). Conversely, corals with horizontal transmission acquire symbionts from the environment with every generation, and this flexibility, which may increase competition between symbionts leading to exploitation of hosts, may promote greater physiological fitness under environmental stress (11, 30, 43, 44). However, both modes of symbiont transmission may be less constrained than predicted, as VT corals acquire novel phylotypes from the environment and HT corals inherit phylotypes more frequently than expected (45–47), suggesting that both could both benefit from dynamically adjusting phylotype concentrations within their tissues (symbiont shuffling) and acquiring novel phylotypes (symbiont switching) to increase thermal resilience. We show that higher specificity of association increases resilience in HT corals. This may be due to stronger cooperation with host-specific phylotypes rather than to potentially weaker cooperation with thermotolerant generalist phylotypes, whose interests may be poorly aligned with those of their hosts (11). However, the thermal resilience benefit resulting from associations with host-specific phylotypes is not due to the greater thermotolerance of this group, as the number of coral partners a phylotype associates with is unrelated to thermotolerance. Among VT corals, but not HT corals, an increased number of phylotypes is correlated with a greater bleaching response, similar to increased environmental susceptibility previously observed among generalist corals (32, 33).

The strategy of VT corals resembles frontloading of genes in thermally resilient corals, where greater transcription levels for several genes prior to stressful conditions seem to confer tolerance to thermal stress (48). It may not increase resiliency of HT corals to associate *a priori* with thermotolerant phylotypes, due to a greater flexibility in symbiont switching or symbiont shifting under elevated temperatures (30, 31). These distinct strategies could be due to contrasting physiological mechanisms involved in the response of the coral to heat stress. For example, Acropora millepora (horizontally transmitting) and Stylophora pistillata (vertically transmitting) experimentally exposed to temperature anomalies showed similar stress responses regarding breakdown of symbiont photosynthetic efficiency and symbiont loss, but differ significantly in the regulation and production of oxidative stress compounds (49).

We evaluated increased thermal resilience of hosts that could be correlated with specific association patterns by examining the global coral-Symbiodiniaceae network of associations during normal, nonstress, conditions. Coral-Symbiodiniaceae interactions are known to change, even if only temporarily, during bleaching and recovery periods (50–52) and may originate from acclimation or adaptation processes related to environmental stress (30, 53, 54), so analysis of these interactions would reveal valuable insights into the thermal resilience of the holobiont. However, these interactions are known for only a fraction of the species and seem to depend on the severity of stress (34, 55) making cross-species comparisons under thermal stress extremely challenging. Furthermore, our analysis should be interpreted within the limitations of the ITS2 Symbiodiniaceae marker, which, due to high intragenomic variability (IGV), has shown poor resolution to distinguish multiple host-specific Symbiodiniaceae lineages recently identified by higher-resolution genetic markers (1, 2, 16, 56). Nevertheless, the ITS2 marker is the most widely used and has the most extensively characterized thermotolerance of any available data, allowing us to build the most comprehensive data set currently possible. We examined the effects of intragenomic diversity and host specificity on our conclusions by creating a modified network where Symbiodiniaceae potential IGVs were considered host specific, which eliminated about 25% of the unique associations from our network but did not change the main conclusions of our study. Therefore, our results provide a robust starting point to evaluate the potential benefits in thermal resilience to the holobiont that result from specific association patterns.

Our findings indicate that the potential benefit of increased frequency of interaction with thermotolerant phylotypes it is not universally extensible to all coral species, but it is a strategy that can only be used by VT corals to increase thermal resilience. Adding bleaching and recovery symbiont assemblage data should further illuminate strategies used by HT and VT corals under thermal stress. However, this current work has the potential to inform strategies for conservation and restoration of reef ecosystems under climate change, particularly for approaches that look to increase bleaching resilience through greater symbiont thermal tolerance prior to thermal stress.

## MATERIALS AND METHODS

### Frequency-weighted bipartite network analysis.

A frequency-of-interaction matrix was built between 152 coral species and 385 Symbiodiniaceae phylotypes, encompassing 1,283 unique interactions and 15,915 total interaction records observed under nonbleaching conditions following Swain et al. (8) (see Tables S2 andS3 and section S.1 in reference 35). Species traits were assigned to both partners, namely symbiont mode of transmission (horizontally transmitted [HT] or vertically transmitted [VT]), ocean biogeography (Atlantic or Indo-Pacific) and coral life history strategy (competitive, weedy, stress tolerant, or generalist; see Tables S4 and S5 in reference 35). Each record of association is one observation of a single Symbiodiniaceae phylotype *in hospite* (characterized using the internal transcribed spacer 2 of the ribosomal RNA nuclear gene [ITS2 rDNA]; see section S.1 in reference 35) with an individual coral colony and does not include repeated sampling of the same phylotype within the same coral colony. However, different phylotypes cooccurring in the same coral colony are recorded as unique association observations. Recently, the ITS2 rDNA marker has been found to be limited in its ability to resolve Symbiodiniaceae species because its multiple copies per genome may not be completely homogenized by concerted evolution, resulting in intragenomic diversity, which convolutes species delineations and provides limited resolution of high-level host specificity (1, 2, 16, 56). Nevertheless, ITS2 is the most widely used Symbiodiniaceae genetic marker, and functional characterizations of Symbiodiniaceae lineages, such as thermotolerance, have been performed for ITS2 symbiont types (18). We examined the effects of intragenomic diversity and host specificity on our conclusions by creating a modified network where Symbiodiniaceae potential intragenomic variants were considered host specific, which affected symbiont nodes and their links to other coral hosts (see “Sensitivity analysis”). The coral-symbiont matrix of interactions was represented as a bipartite network, which comprises two types of nodes (coral species and symbiont phylotypes), and only connections (edges or links) between different types of nodes are allowed. We include interaction frequency between partners (frequency-weighted node connections) to evaluate which associations are more significant to the symbiosis, instead of assuming that all associations are equally important, by assessing presence or absence of interactions. Therefore, link strength, a measure of the frequency of interaction with each symbiont, was weighted by proportion rather than by the absolute number of associations in the data set (which differed significantly between coral species), such that the weights of all links originating from a coral node summed to one. Networks were visualized in Cytoscape using a link-weighted, force-directed algorithm that positions highly connected nodes together such that the distance between nodes decreases as the relative frequency of observation increases (see section S.2 in reference 35).

We assessed network structural heterogeneity and patterns of interaction between corals and their symbionts through metrics of connectance, asymmetry, centrality, modularity, and beta diversity (see section S.3 in reference 35). Centrality (scaled to a maximum of 1) measures the influence of each node within the network, capturing how highly connected each node is and how closely connected it is to high-centrality nodes. Because there is no well-established centrality algorithm suited to bipartite networks, our eigenvector centrality calculations were based on a one-mode projection of the network and weighted by link strength (implemented in the *igraph* R package [57]). Network modularity (calculated using the *bipartite* R package [58]) was used to evaluate whether coral-symbiont interactions are structured into groups or modules, (i.e., do within-module species share a higher proportion of interactions than species in different modules?) and to assess whether phylotypes establish strong links mostly within or across modules. Each phylotype was assigned two scores after Olesen et al. (59), a “within-module degree,” *z* (the standardized number of links to other corals within the same module), and their “among-module connectance,” *c* (the level to which the phylotype is linked to species in other modules) (59). Boundaries indicating significantly high *c* and *z* scores were based on 1,000 random permutations of the network, with the 95th percentile used to establish significance (0.6 for *c*, 2 for *z* [59]). Finally, we calculated the beta diversity between each pair of coral species to characterize differences in phylotype composition and phylogenetic relatedness between coral species, using two metrics, Bray-Curtis dissimilarity and UniFrac distance (60) (see also section S.3 in reference 35).

### Phylogenetic analyses.

Phylogenies for coral species and Symbiodiniaceae phylotypes were created by adapting previously published trees to the taxon sets in this study (see section S.4 in reference 35). Correlations between node traits were assessed using phylogenetic comparative methods to evaluate the effects of nonindependence among species and phylotypes due to evolutionary relatedness (61). Three types of analysis were used, namely phylogenetic logistic regression (PLR; correlates binary and continuous characters; see Table S6 in reference 35), phylogenetic analysis of variance (PANOVA; correlates continuous characters across multiple groups; see also Table S7 in reference 35), or phylogenetic generalized least-squares regression (PGLS; correlates continuous characters within two groups; see also Table S8 in reference 35). The extent to which phylogenetic relatedness structures the network for different traits was evaluated by calculating phylogenetic signal, Pagel’s λ, which is a measure of phylogenetic dependence of the data (62) that ranges between 0 (phylogenetic independence) and 1 (traits covary in direct proportion to their shared evolutionary history; see Table S9 in reference 35). We also tested the distributions of within-module and between-module phylogenetic distances for both coral and Symbiodiniaceae nodes. Significance was assessed via a nonparametric *t* test with comparison to 10,000 permutations of the randomized data set.

### Richness, frequency of interaction, and specificity.

Frequency-weighted networks can be characterized by (i) the number of links between different partners (richness), (ii) the strength of the link between each coral-phylotype pair (frequency of interaction), and (iii) the relative strength of the link of a coral-phylotype pair relative to all link strengths of that phylotype (specificity *d′*) (see section S.5 in reference 35). Briefly, coral node richness was calculated following Swain et al. (8), who identified biases when determining richness from direct counting of the number of unique phylotypes due to highly uneven sampling across coral species and demonstrated that those biases could be mitigated through rarefaction. We used this approach to calculate rarefied richness for coral species with at least 10 association records, as their Symbiodiniaceae assemblages are likely to be better sampled. Because the rank ordering of richness between Symbiodiniaceae assemblages may differ depending on the sample size chosen for rarefaction, we calculated rarefied richness at different sampling sizes, namely, rarefaction depths of 10, 15, and 20 interaction records per coral (RD_10_, RD_15_, and RD_20_, respectively), resulting in data subsets with 152, 123, and 105 coral species, respectively. Frequency of interaction was calculated as the number of interactions of a coral-phylotype pair relative to all the interactions between that coral and other phylotypes. Finally, specificity *d′*, which ranges between 0 (generalist coral interacting with generalist phylotypes) and 1 (specialist coral interacting with a specialist phylotype) was calculated for each partner individually as in Blüthgen (26).

### Coral thermal resilience (taxon-BRI) and phylotype thermotolerance (TT).

All 152 coral species nodes were assigned thermal resilience scores measured as the species-specific bleaching response index (taxon-BRI [17]; see also Table S4 in reference 35). Taxon-BRI ranges from 0 to 100, where 0 corresponds to absence of bleaching response under thermal stress (high thermal resilience), and 100 corresponds to high bleaching and mortality (low thermal resilience). Several traits were tested against taxon-BRI of corals in the entire network or between corals with bleaching susceptibility in the top third (high BRI, *n* = 56 species, 39 HT and 17 VT) and the bottom third (low BRI, *n* = 56 species, 40 HT and 16 VT) of species (see section S.6 in reference 35).

Thermotolerance (TT) of Symbiodiniaceae (ITS2 phylotype-specific thermotolerance index [18]) was known for only a subset of phylotype nodes (73 out of 385), which nevertheless were responsible for 75.7% (12,049 out of 15,915) of all interaction records. Phylotypes with missing TT data were not included in TT-focused analyses. TT was measured as phylotype-specific thermotolerance scores describing a percentile consensus ranking of relative thermotolerance ranging from 0 to 100 (thermosensitive to thermotolerant [18]; see also Table S5 in reference 35). The thermotolerance of Symbiodiniaceae assemblages of individual coral species was calculated as the interaction frequency-weighted mean of thermotolerance scores (mean-TT), and thermotolerance diversity of that assemblage was calculated as the standard error of the frequency-weighted mean of thermotolerance scores (stderror mean-TT). Frequency of interaction with phylotypes with known thermotolerance was calculated for prominent phylotypes (e.g., thermotolerant D1-4 and thermosensitive C3) and for phylotypes grouped by their relative thermotolerance (5 bins with increasing thermotolerance percentile cutoffs, as follows: TT ≤ 10, 10 < TT ≤ 20, 20 < TT ≤ 30, 30 < TT ≤ 40, and TT > 40; see section S.6 and Table S10 in reference 35).

### Sensitivity analysis.

We evaluated the sensitivity of our results to the diversity of symbiont phylotypes characterized using ITS2, which may artificially affect our analyses due to intragenomic variants (IGVs) and low resolution in distinguishing host-specific lineages (1). We constructed a new matrix of coral-phylotype interactions implementing the concept of metahaplotypes (*sensu* Smith et al. [56]), where potential IGVs cooccurring with ancestral phylotypes in a single host were treated as a single host-specific Symbiodiniaceae lineage metahaplotype. Potential IGVs without evidence of ancestral cooccurrence were eliminated from the data set. We used this modified data set to recalculate rarefied richness and specificity *d′*, and to construct a new bipartite network to reexamine the critical findings of the main analysis (see section S.7 and Tables S11 and S12 in reference 35). Furthermore, we ran analyses to evaluate the effect of unequal sampling intensity across coral species (measured as number of interaction records for each species). We first tested the possibility that the metrics used in this study (in particular rarefied richness) could be confounded by sampling intensity by running phylogenetically corrected analysis between sampling intensity and different metrics, including taxon-BRI. We then repeated the phylogenetically corrected analyses in this study using three subsets of the main data set with increasing sampling accuracy (RD_10_, RD_15_, and RD_20_; see section S.7 and Table S13 in reference 35).

10.1128/mSystems.00266-21.10 FIG S10 Module detection in weighted bipartite networks produces unstable results. Our implementation of Beckett’s algorithm is based on optimization via 10^6^ random stitches in module organization, resulting in high variation in the number of detected modules (*x* axis), but relatively minor variation in the modularity index (*Q*) of the organization. We ran the algorithm 1,000 times, and to simplify our analyses, we selected the more modular of the two outputs with the minimum number of modules (7), highlighted with the red circle. Download FIG S10, PDF file, 0.1 MB. Copyright © 2021 Swain et al. 2021 Swain et al. https://creativecommons.org/licenses/by/4.0/ This content is distributed under the terms of the Creative Commons Attribution 4.0 International license.
