# Supplementary figures and images for: A Phylogeny-Informed Analysis of the Global Coral-Symbiodiniaceae Interaction Network Reveals that Traits Correlated with Thermal Bleaching Are Specific to Symbiont Transmission Mode
Source: mSystems. 2021 May 4;6(3):e00266-21. doi: 10.1128/mSystems.00266-21 (PMC8269218; doi:10.1128/mSystems.00266-21)

FigS1

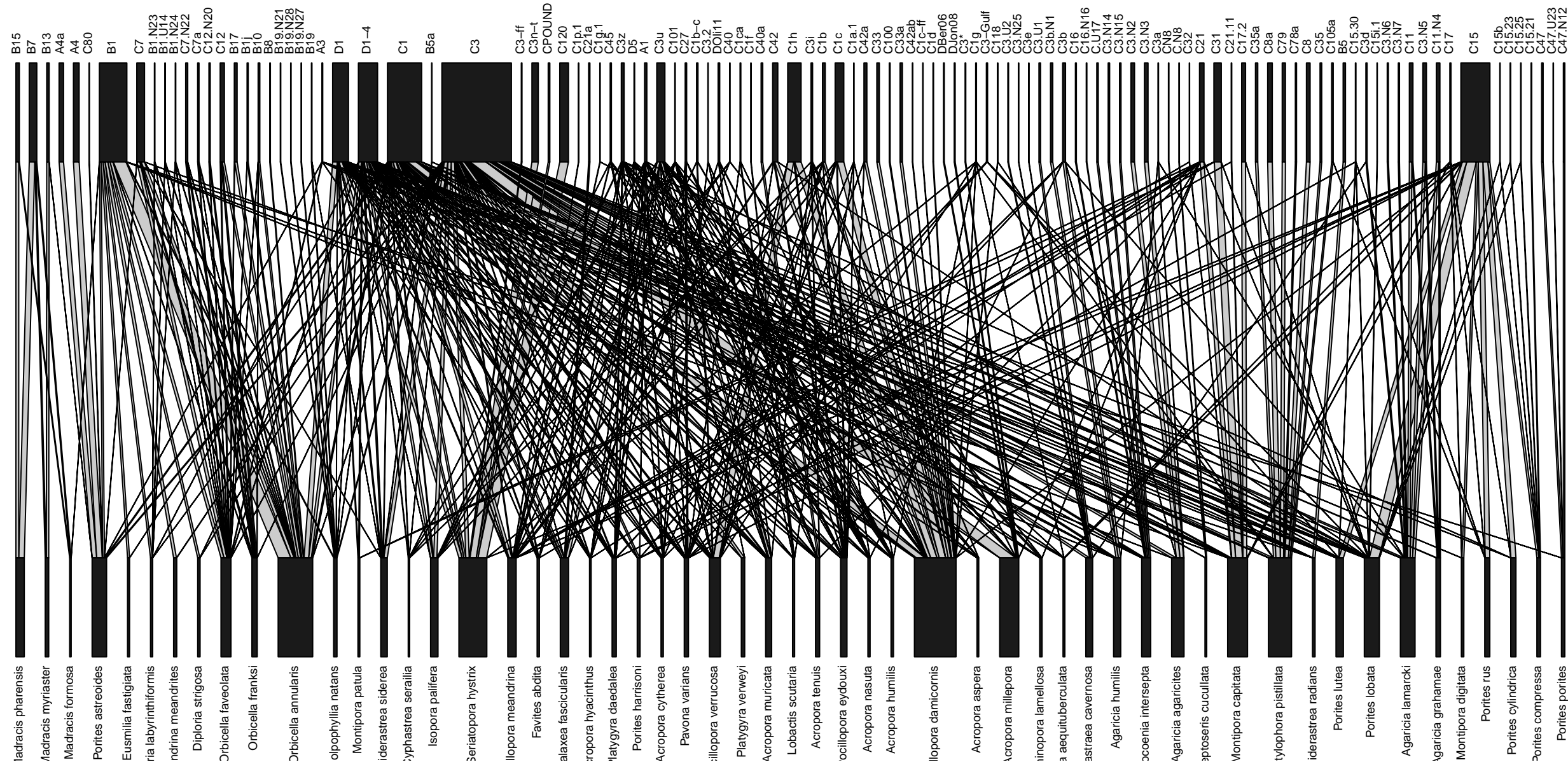

Supplement: FIG S1 [file msystems.00266-21-sf001.pdf]

FigS3

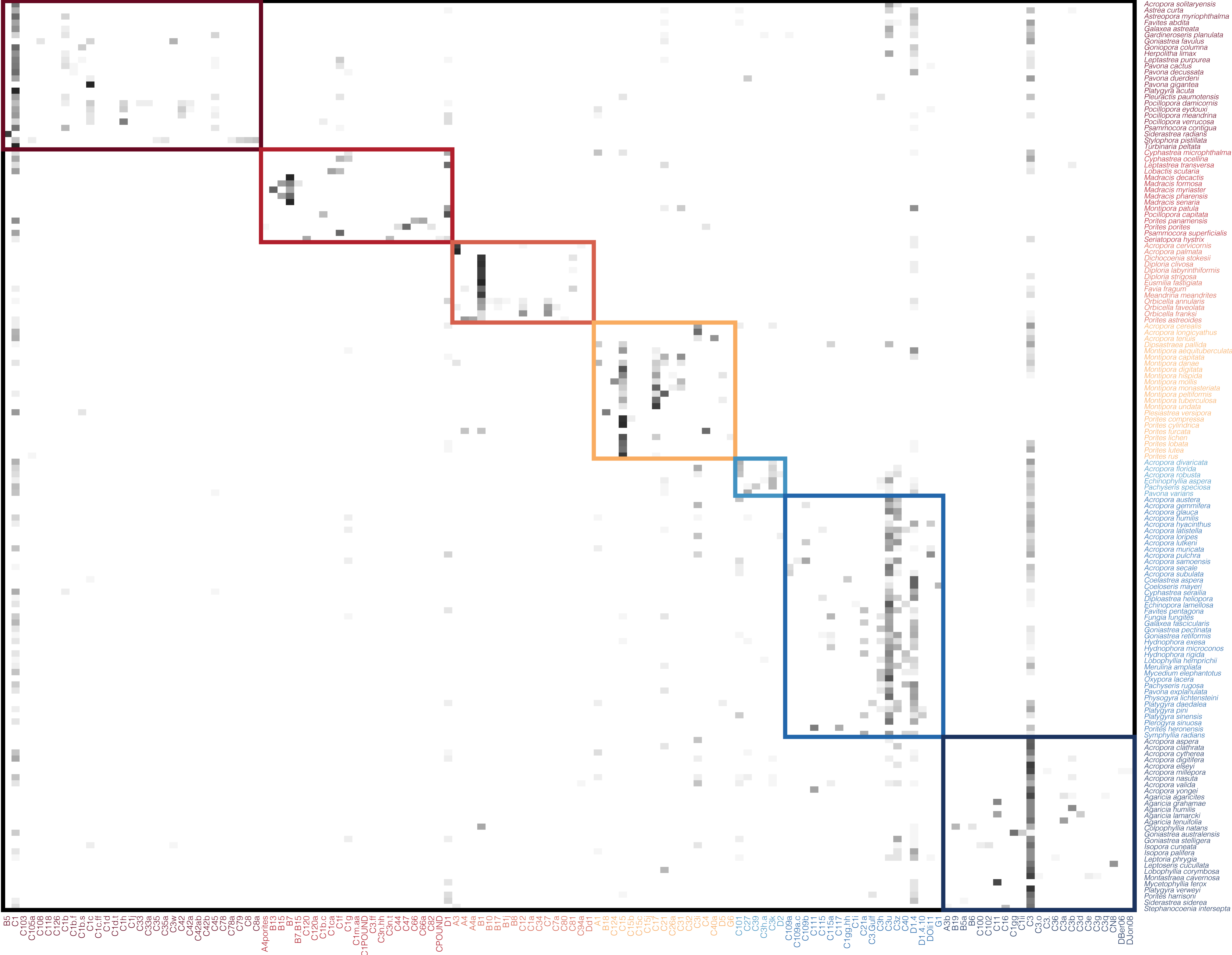

Supplement: FIG S3 [file msystems.00266-21-sf003.pdf]

FigS4

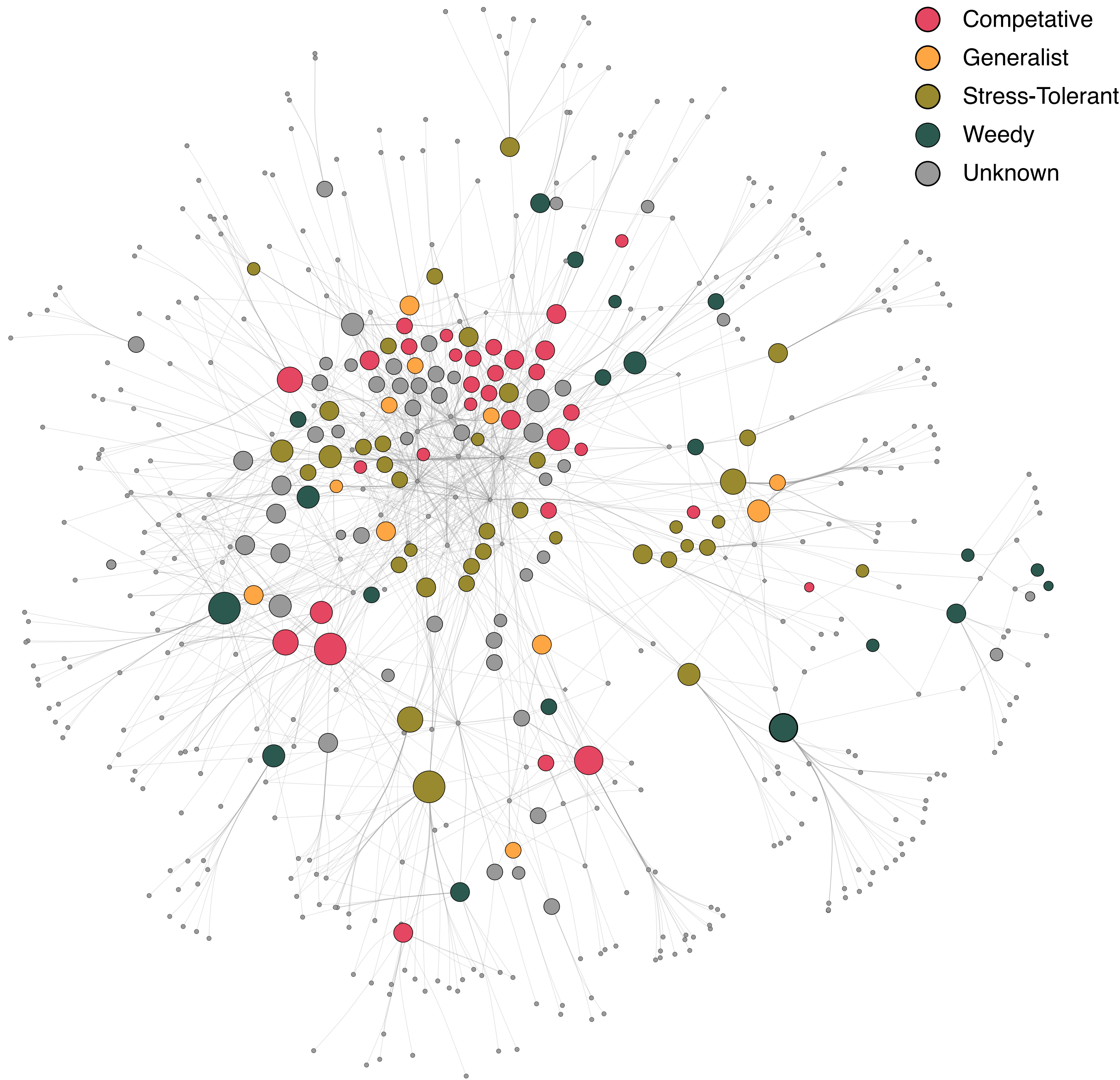

Supplement: FIG S4 [file msystems.00266-21-sf004.pdf]

FigS5

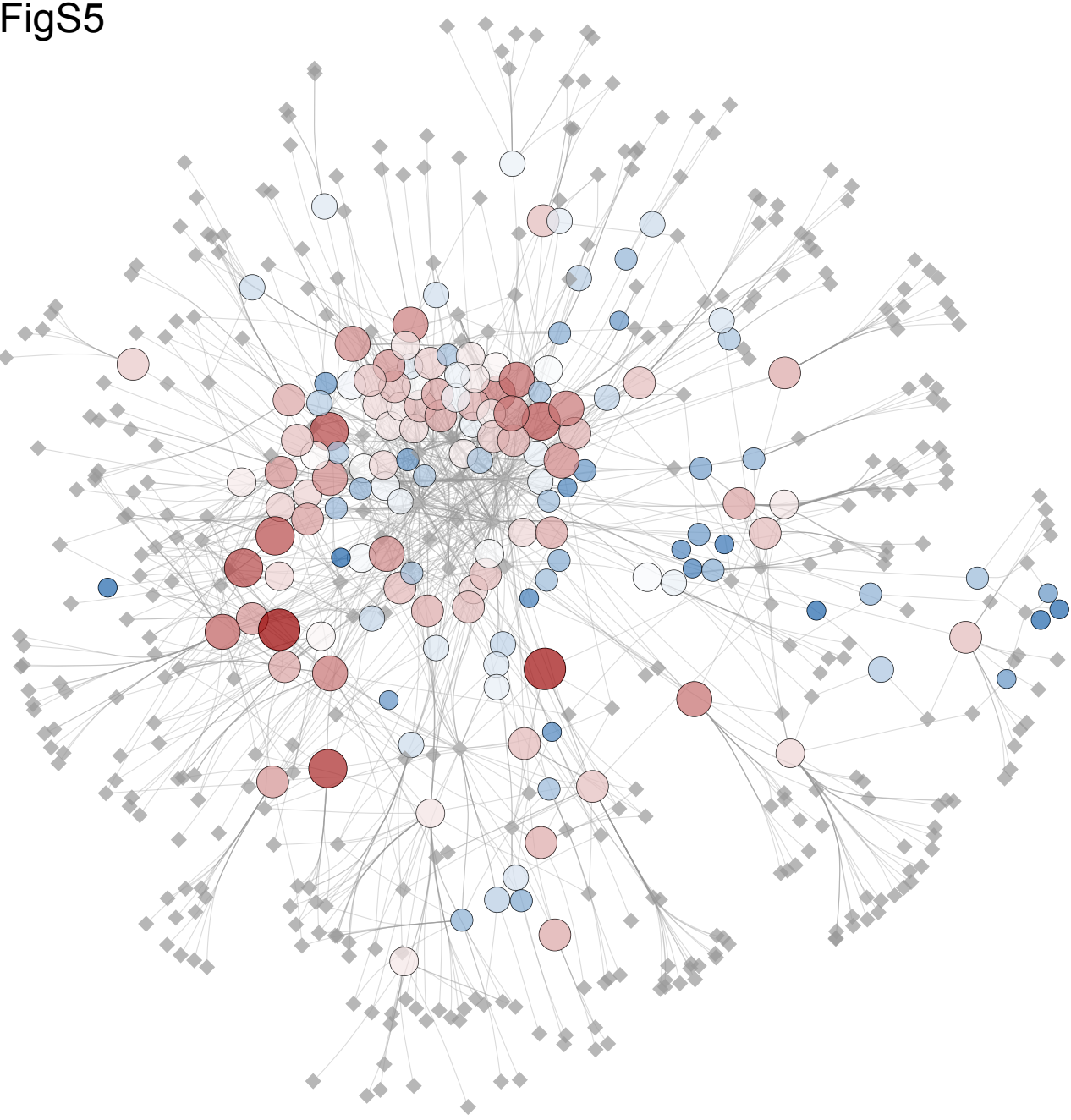

Rarefied Richness (RD10)

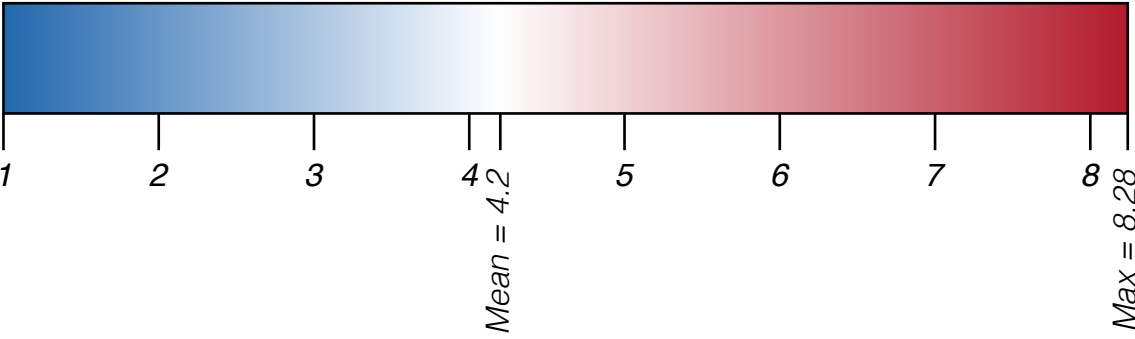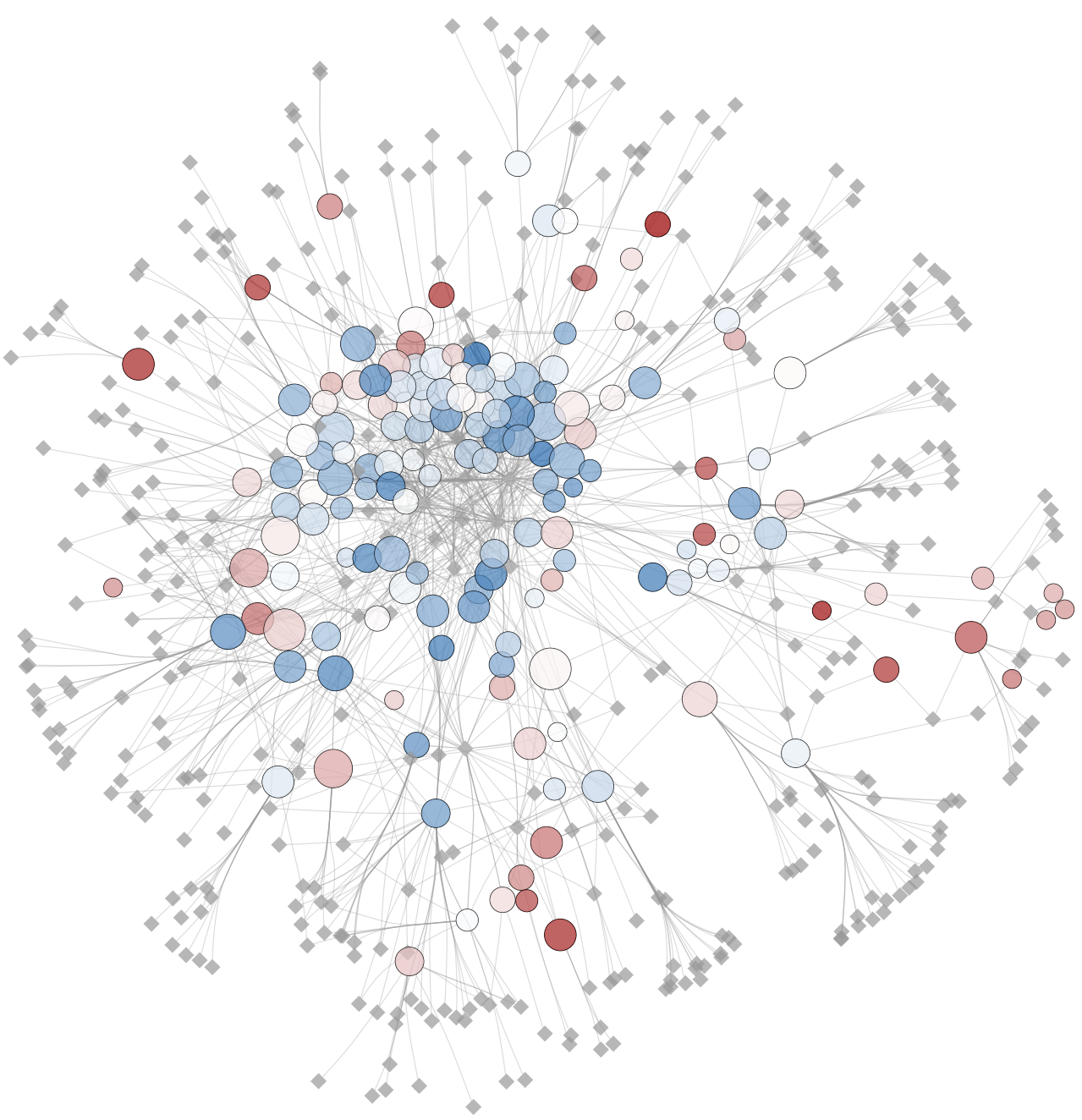

Specificity  $d'$

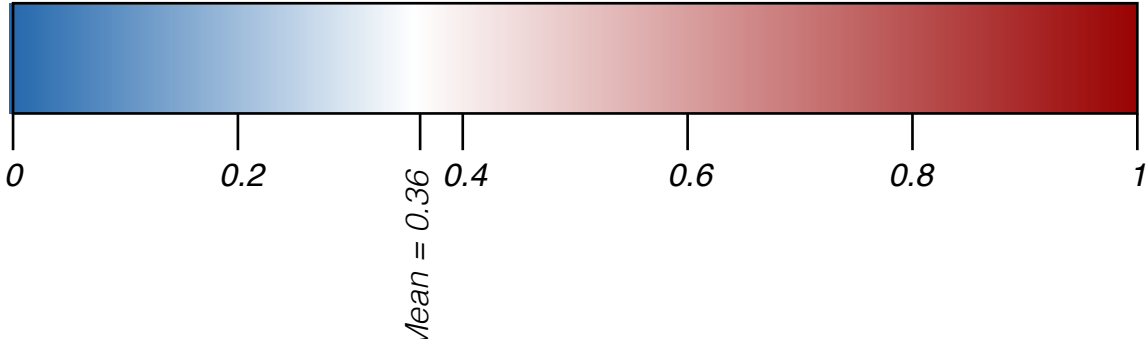

Supplement: FIG S5 [file msystems.00266-21-sf005.pdf]

FigS6

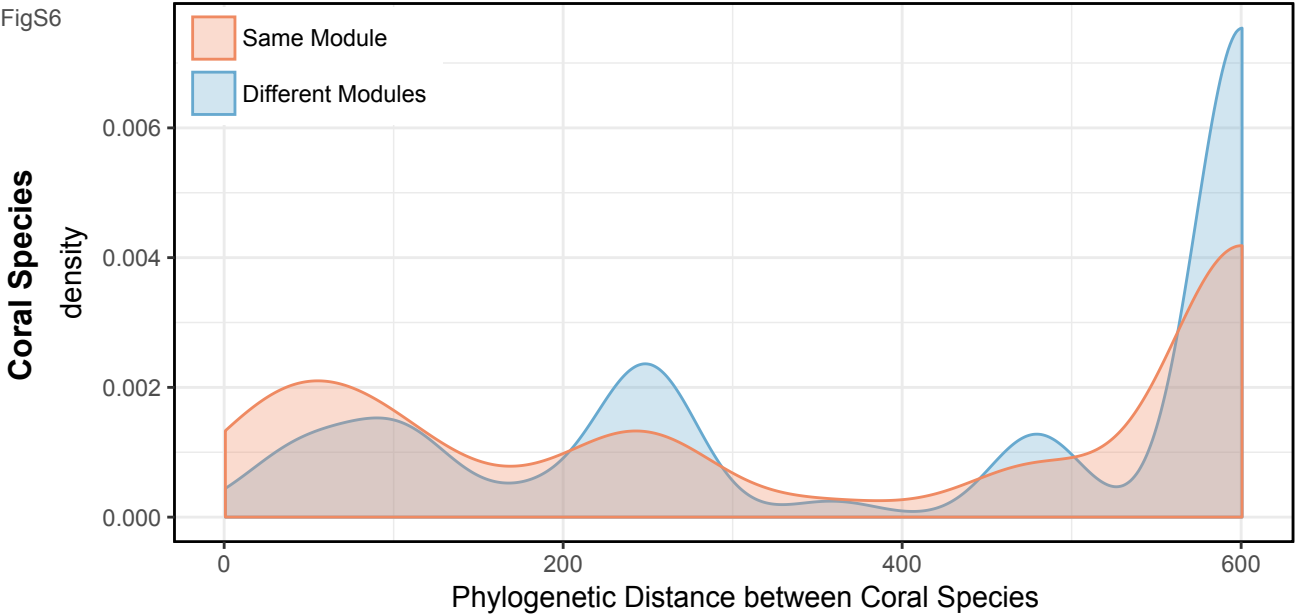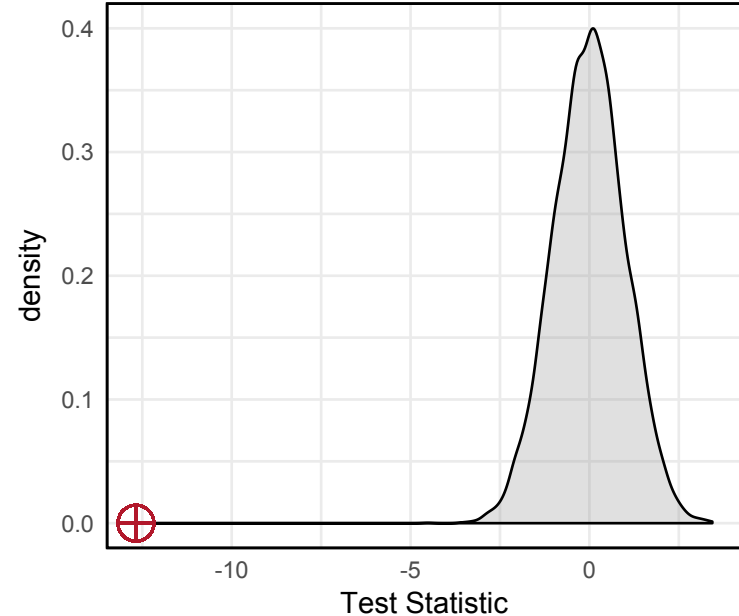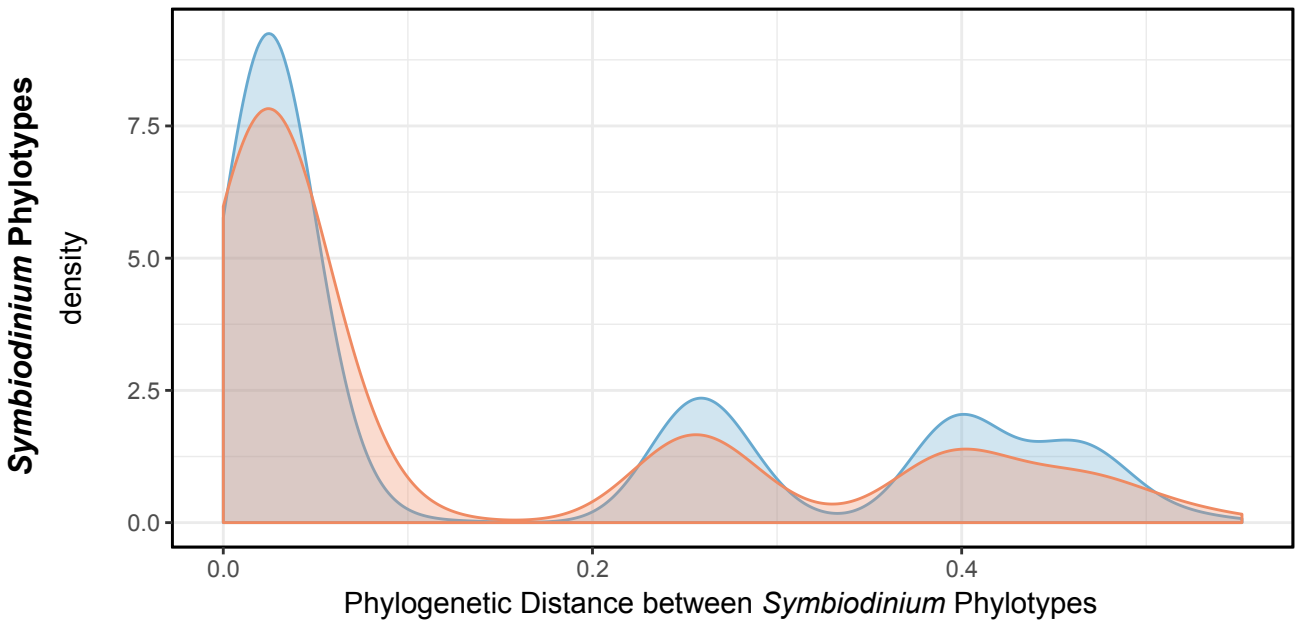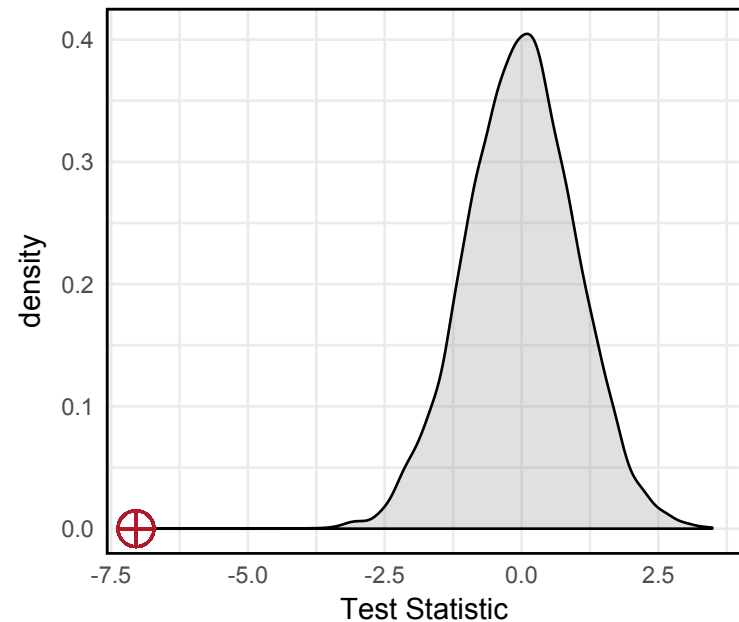

Supplement: FIG S6 [file msystems.00266-21-sf006.pdf]

FigS7

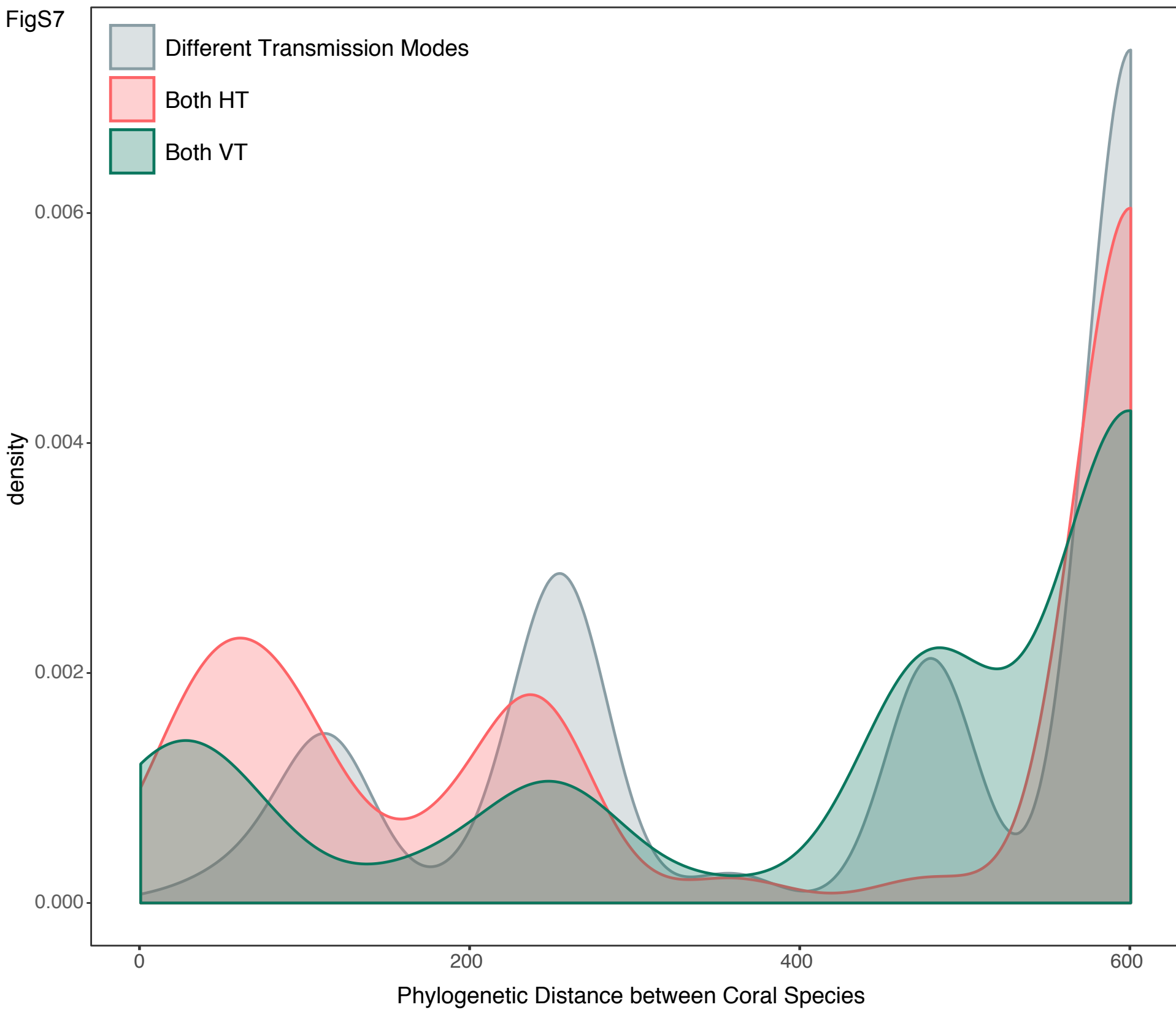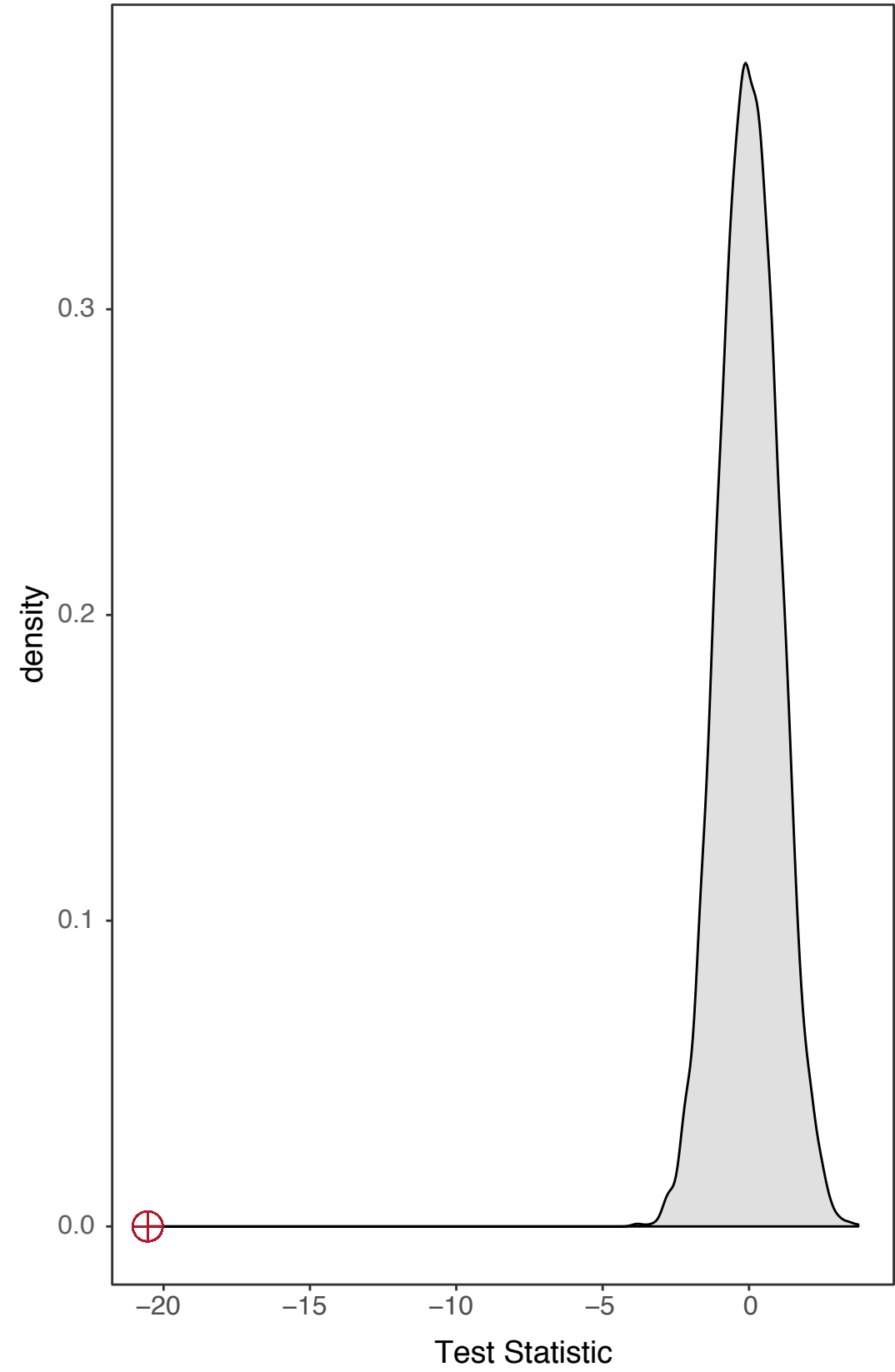

Supplement: FIG S7 [file msystems.00266-21-sf007.pdf]

FigS8

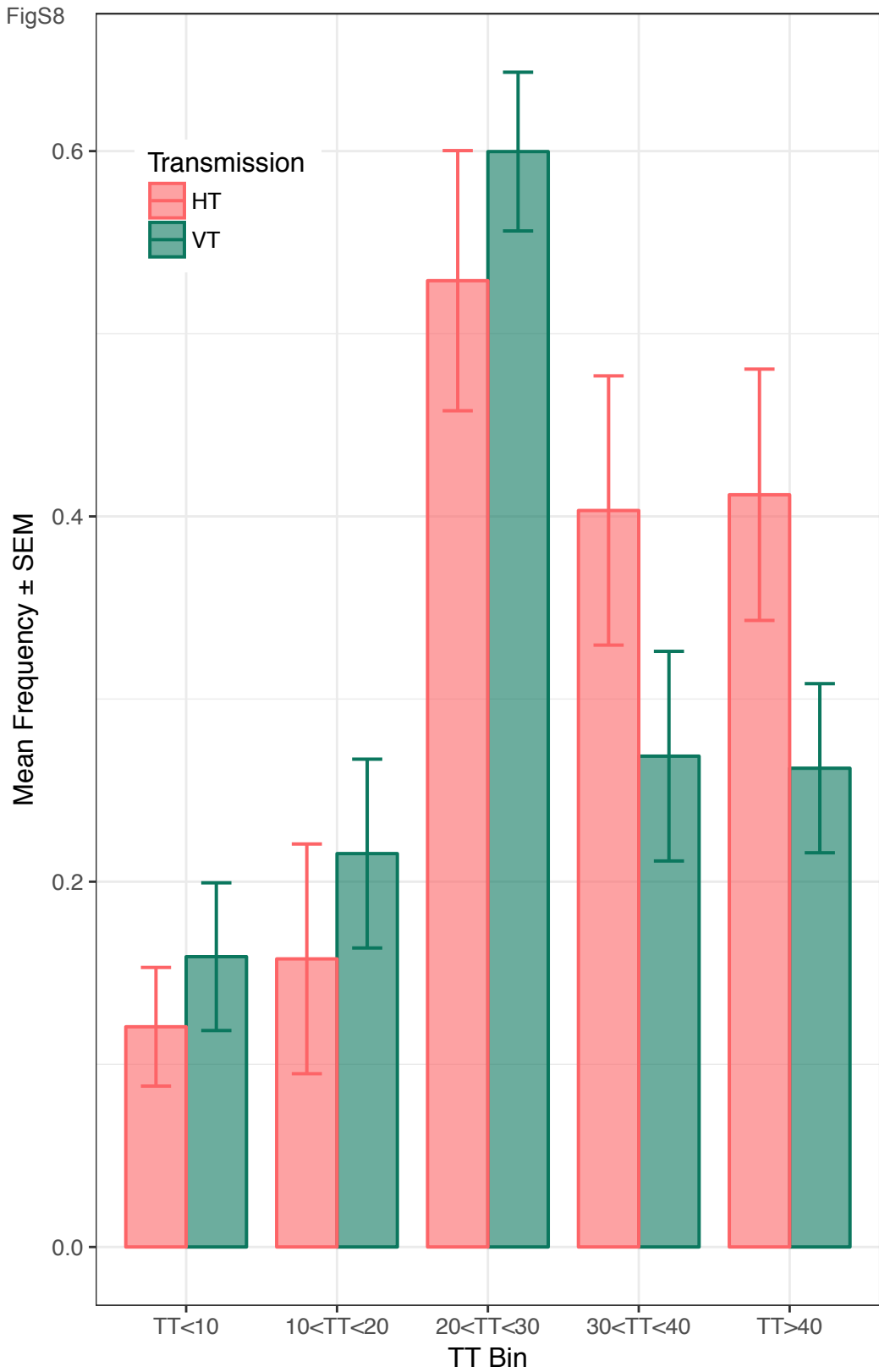

Supplement: FIG S8 [file msystems.00266-21-sf008.pdf]

FigS9

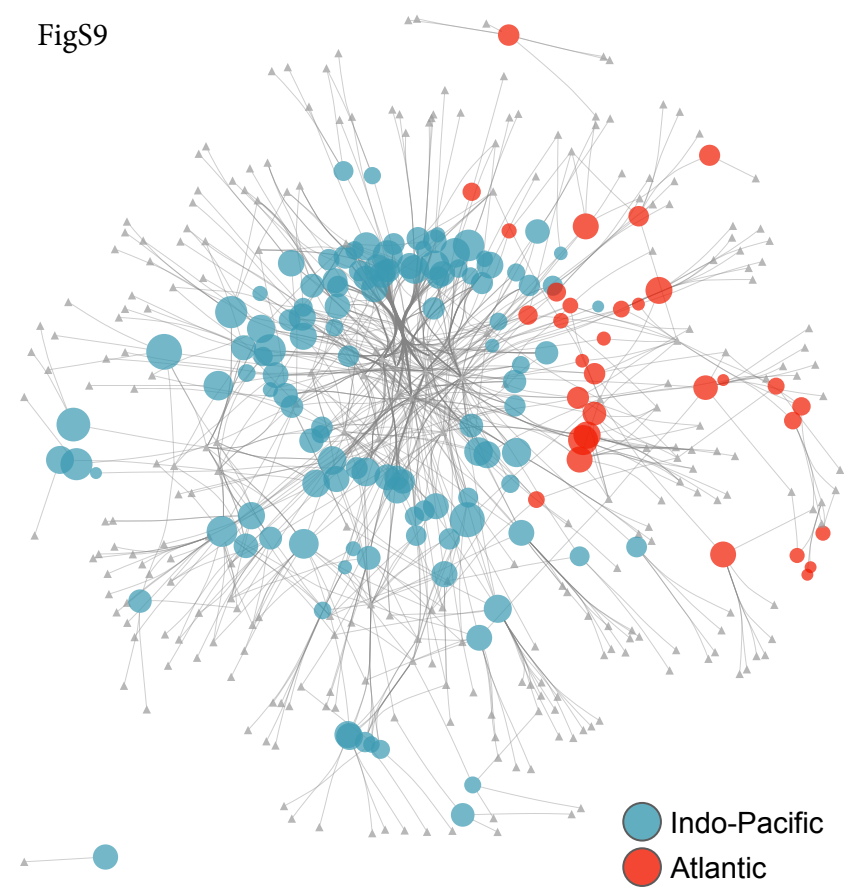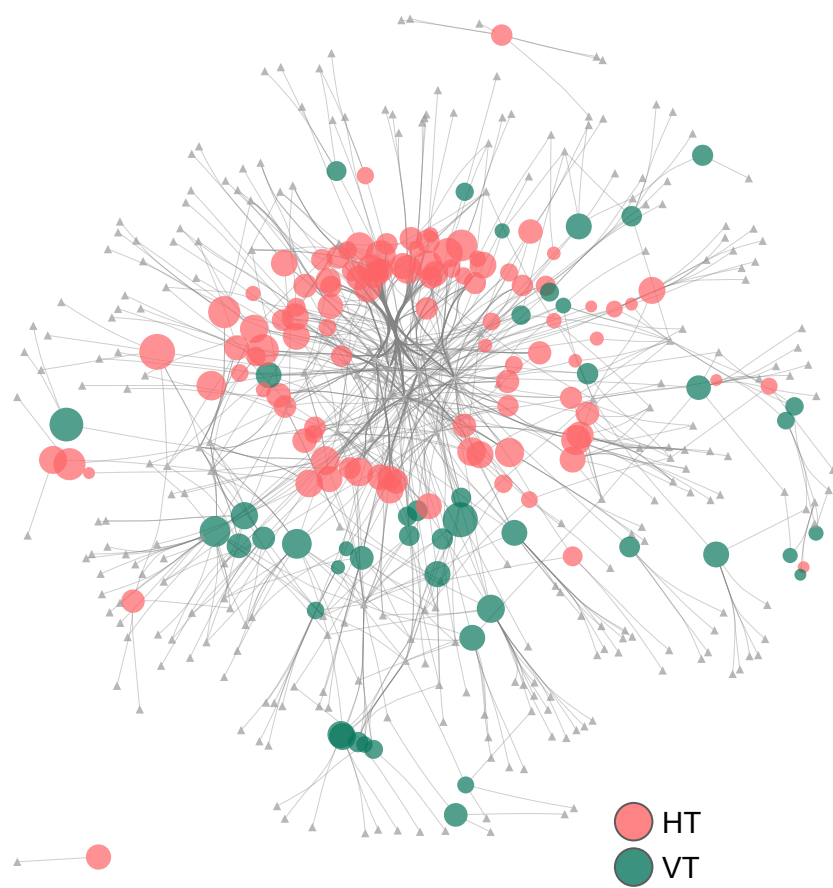

Supplement: FIG S9 [file msystems.00266-21-sf009.pdf]

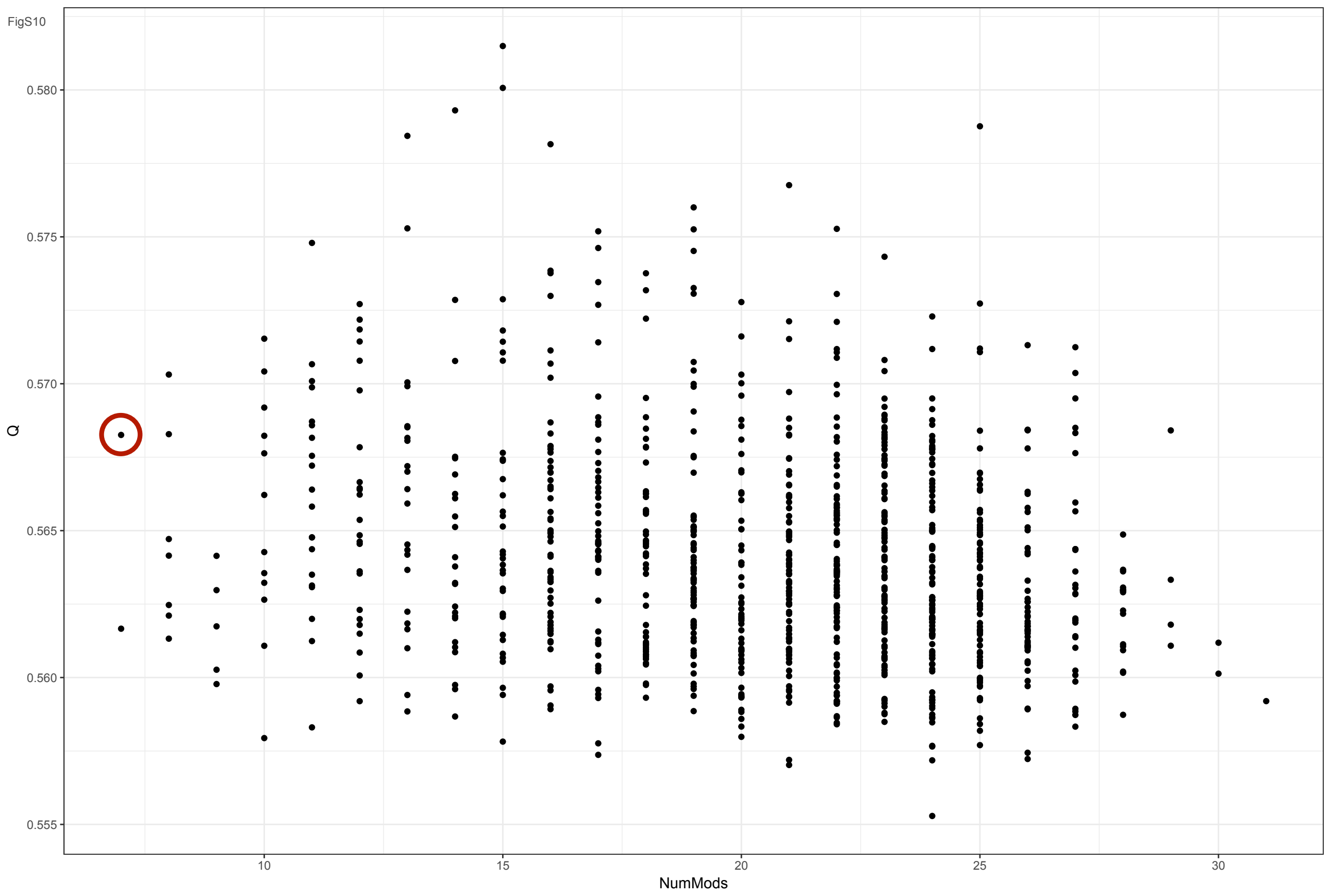

Supplement: FIG S10 [file msystems.00266-21-sf010.pdf]
