# Supplementary material for: A Phylogeny-Informed Analysis of the Global Coral-Symbiodiniaceae Interaction Network Reveals that Traits Correlated with Thermal Bleaching Are Specific to Symbiont Transmission Mode
Source: mSystems. 2021 May 4;6(3):e00266-21. doi: 10.1128/mSystems.00266-21 (PMC8269218; doi:10.1128/mSystems.00266-21)
Supplement: FIG S2 [file msystems.00266-21-sf002.pdf]

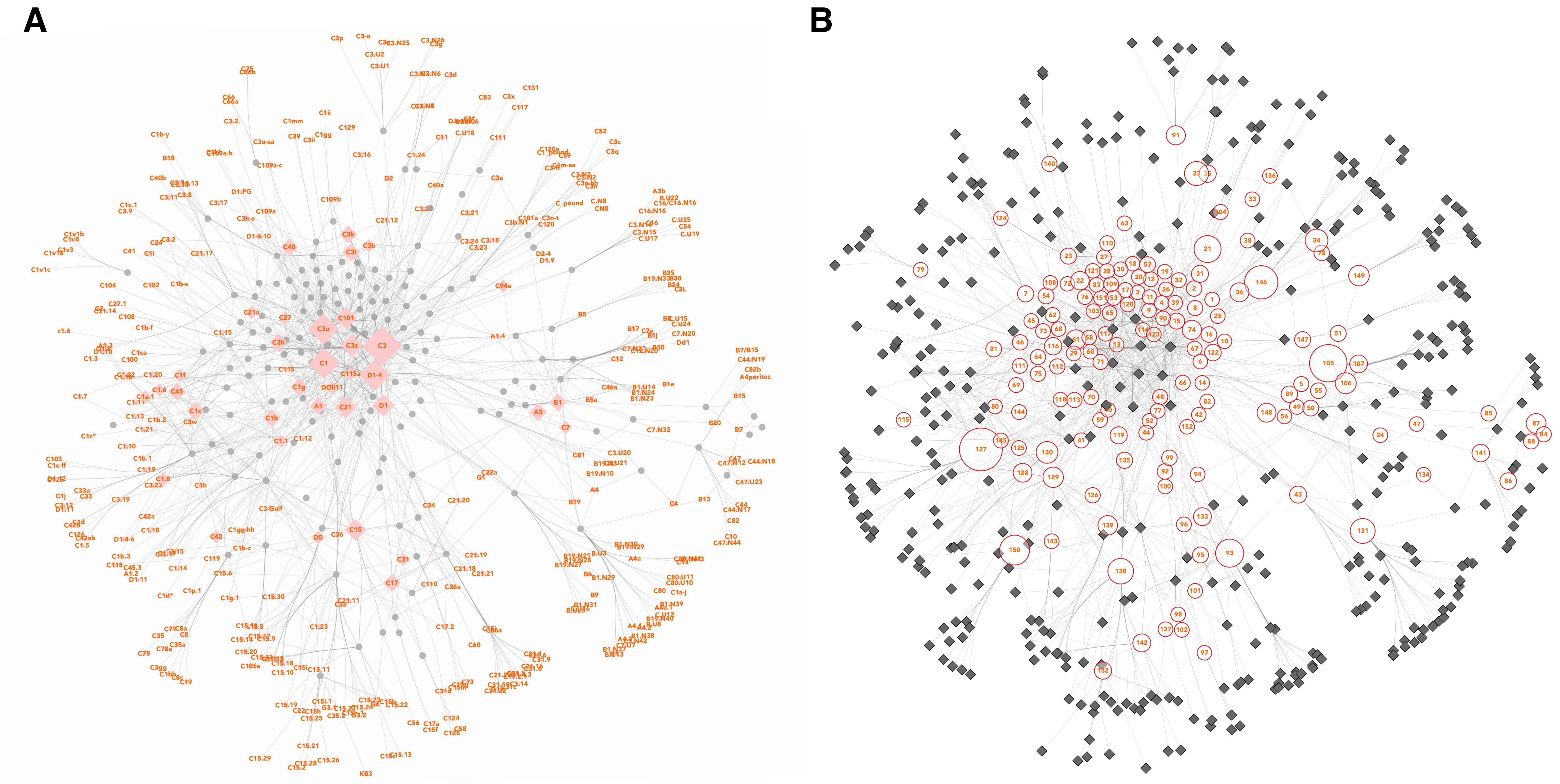

1	*Acropora abrotanoides*	52	*Dipsastraea pallida*	103	*Mycedium elephantotus*
2	*Acropora aspera*	53	*Echinophyllia aspera*	104	*Mycetophyllia ferox*
3	*Acropora austera*	54	*Echinopora lamellosa*	105	*Orbicella annularis*
4	*Acropora cerealis*	55	*Eusmilia fastigiata*	106	*Orbicella faveolata*
5	*Acropora cervicornis*	56	*Favia fragum*	107	*Orbicella franksi*
6	*Acropora clathrata*	57	*Favites abdita*	108	*Oxypora lacera*
7	*Acropora cytherea*	58	*Favites pentagona*	109	*Pachyseris rugosa*
8	*Acropora digitifera*	59	*Fungia fungites*	110	*Pachyseris speciosa*
9	*Acropora divaricata*	60	*Galaxea astreata*	111	*Pavona cactus*
10	*Acropora elseyi*	61	*Galaxea fascicularis*	112	*Pavona decussata*
11	*Acropora florida*	62	*Gardineroseris planulata*	113	*Pavona duerdeni*
12	*Acropora gemmifera*	63	*Goniastrea australensis*	114	*Pavona explanulata*
13	*Acropora glauca*	64	*Goniastrea favulus*	115	*Pavona gigantea*
14	*Acropora grandis*	65	*Goniastrea pectinata*	116	*Pavona varians*
15	*Acropora humilis*	66	*Goniastrea retiformis*	117	*Physogyra lichtensteini*
16	*Acropora hyacinthus*	67	*Goniastrea stelligera*	118	*Platygyra acuta*
17	*Acropora latistella*	68	*Goniopora columna*	119	*Platygyra daedalea*
18	*Acropora longicyathus*	69	*Herpolitha limax*	120	*Platygyra pini*
19	*Acropora loripes*	70	*Hydnophora exesa*	121	*Platygyra sinensis*
20	*Acropora lutkeni*	71	*Hydnophora microconos*	122	*Platygyra verweyi*
21	*Acropora millepora*	72	*Hydnophora rigida*	123	*Plerogyra sinuosa*
22	*Acropora muricata*	73	*Isopora cuneata*	124	*Plesiastrea versipora*
23	*Acropora nasuta*	74	*Isopora palifera*	125	*Pleuractis paumotensis*
24	*Acropora palmata*	75	*Leptastrea purpurea*	126	*Pocillopora capitata*
25	*Acropora pulchra*	76	*Leptastrea transversa*	127	*Pocillopora damicornis*
26	*Acropora robusta*	77	*Leptoria phrygia*	128	*Pocillopora eydouxi*
27	*Acropora samoensis*	78	*Leptoseris cucullata*	129	*Pocillopora meandrina*
28	*Acropora secale*	79	*Leptoseris papyracea*	130	*Pocillopora verrucosa*
29	*Acropora solitaryensis*	80	*Lithophyllon concinna*	131	*Porites astreoides*
30	*Acropora subulata*	81	*Lobactis scutaria*	132	*Porites compressa*
31	*Acropora tenuis*	82	*Lobophyllia corymbosa*	133	*Porites cylindrica*
32	*Acropora valida*	83	*Lobophyllia hemprichii*	134	*Porites furcata*
33	*Acropora yongei*	84	*Madracis decactis*	135	*Porites harrisoni*
34	*Agaricia agaricites*	85	*Madracis formosa*	136	*Porites heronensis*
35	*Agaricia grahamae*	86	*Madracis myriaster*	137	*Porites lichen*
36	*Agaricia humilis*	87	*Madracis pharensis*	138	*Porites lobata*
37	*Agaricia lamarcki*	88	*Madracis senaria*	139	*Porites lutea*
38	*Agaricia tenuifolia*	89	*Meandrina meandrites*	140	*Porites panamensis*
39	*Astrea curta*	90	*Merulina ampliata*	141	*Porites porites*
40	*Astreopora myriophthalma*	91	*Montastraea cavernosa*	142	*Porites rus*
41	*Coelastrea aspera*	92	*Montipora aequituberculata*	143	*Porites solida*
42	*Coeloseris mayeri*	93	*Montipora capitata*	144	*Psammocora contigua*
43	*Colpophyllia natans*	94	*Montipora danae*	145	*Psammocora superficialis*
44	*Cyphastrea microphthalma*	95	*Montipora digitata*	146	*Seriatopora hystrix*
45	*Cyphastrea ocellina*	96	*Montipora hispida*	147	*Siderastrea radians*
46	*Cyphastrea serailia*	97	*Montipora mollis*	148	*Siderastrea siderea*
47	*Dichocoenia stokesii*	98	*Montipora monasteriata*	149	*Stephanocoenia intersepta*
48	*Diploastrea heliopora*	99	*Montipora patula*	150	*Stylophora pistillata*
49	*Diploria clivosa*	100	*Montipora peltiformis*	151	*Symphyllia radians*
50	*Diploria labyrinthiformis*	101	*Montipora tuberculosa*	152	*Turbinaria peltata*
51	*Diploria strigosa*	102	*Montipora undata*		
